# Supplementary material for: Comprehensive Meta-Analysis of Futile Recanalization in Acute Ischemic Stroke Patients Undergoing Endovascular Thrombectomy: Prevalence, Factors, and Clinical Outcomes
Source: Life (Basel). 2023 Sep 26;13(10):1965. doi: 10.3390/life13101965 (PMC10608522; doi:10.3390/life13101965)
Supplement: Supplementary file 1 [file life-13-01965-s001.zip › life-2589983-supplementary.pdf]

SUPPLEMENTAL INFORMATION

Comprehensive Meta-Analysis of Futile Recanalization in Acute Ischemic Stroke Patients Undergoing Endovascular Thrombectomy: Prevalence, Factors, and Clinical Outcomes

Authors: Helen Shen, Murray C. Killingsworth and Sonu M. M. Bhaskar*

*Correspondence to: Dr Sonu M. M. Bhaskar, MD PhD
Email: Sonu.Bhaskar@globalhealthneurolab.org / Bhaskar.Sonu@ncvc.go.jp

List of Contents

1. **Search Strategy (Keywords/MeSH Terms)**
   - **a. PubMed Search Strategy**
   - **b. Embase Search Strategy**
   - **c. Cochrane Library Search Strategy**
   - **d. Other Sources**
2. **Supplemental Tables**
   - **a. Supplemental Table S1:** PRISMA-2020 Checklist
   - **b. Supplemental Table S2:** MOOSE Checklist
   - **c. Supplemental Table S3:** Modified Jadad Analysis for Methodological Quality
   - **d. Supplemental Table S4:** Funding Bias Scores for Studies
   - **e. Supplemental Table S5:** Outputs from Egger's Test for Publication Bias for Predictive Indicators
   - **f. Supplemental Table S6:** Summary Effects and Heterogeneity from Meta-analysis of Discrete Predictive Markers and Outcomes Associated with Futile Recanalization
   - **g. Supplemental Table S7:** Summary Effects and Heterogeneity from Meta-analysis of Continuous Predictive Markers Associated with Futile Recanalization
3. **Supplemental Figures**
   - **a. Supplemental Figure S1:** Forest Plots of Discrete Predictive Indicators of Futile Recanalization, Stratified by Occlusion Location (1)
   - **b. Supplemental Figure S2:** Forest Plots of Discrete Predictive Indicators of Futile Recanalization, Stratified by Occlusion Location (2)
   - **c. Supplemental Figure S3:** Forest Plots of Continuous Predictive Indicators of Futile Recanalization, Stratified by Occlusion Location
   - **d. Supplemental Figure S4:** Forest Plots of Discrete Predictive Indicators of Futile Recanalization, Stratified by Study Design (1)
   - **e. Supplemental Figure S5:** Forest Plots of Discrete Predictive Indicators of Futile Recanalization, Stratified by Study Design (2)
   - **f. Supplemental Figure S6:** Forest Plots of Continuous Predictive Indicators of Futile Recanalization, Stratified by Study Design

g. Supplemental Figure S7: Graphs of Egger's Regression Test for Meta-analysis on the Association between Predictive Indicators and Futile Recanalization
h. Supplemental Figure S8: Graphs of Egger's Regression Test for Meta-analysis on the Association between Predictive Indicators and Futile Recanalization (2)
i. Supplemental Figure S9: Graphs of Egger's Regression Test for Meta-analysis on the Association between Clinical Outcomes and Futile Recanalization
j. Supplemental Figure S10: Sensitivity Analysis on Association between Predictive Indicators and Futile Recanalization (1)
k. Supplemental Figure S11: Sensitivity Analysis on Association between Predictive Indicators and Futile Recanalization (2)
l. Supplemental Figure S12: Sensitivity Analysis on Association between Predictive Indicators and/or Outcomes and Futile Recanalization

1. Search Strategy (Keywords/MeSH Terms)

a. PubMed Search Strategy
Search Query: (("stroke"[MeSH Terms] OR "ischemic attack, transient"[MeSH Terms] OR "ischaemic attack transient"[Text Word] OR "cerebrovascular disorders"[MeSH Terms] OR "brain ischemia"[MeSH Terms] OR "brain ischaemia"[Title/Abstract]) AND ("thrombectomy"[MeSH Terms] OR "endovascular procedure*"[MeSH Terms] OR "reperfusion"[MeSH Terms] OR "recanalization"[Title/Abstract] OR "recanalisation"[Title/Abstract]) AND ("futile recanalization"[Title/Abstract] OR "futile recanalisation"[Title/Abstract] OR "failed recanalization"[Title/Abstract] OR "failed recanalisation"[Title/Abstract] OR "complete recanalization"[Title/Abstract] OR "complete recanalisation"[Title/Abstract] OR "partial recanalization"[Title/Abstract] OR "partial recanalisation"[Title/Abstract])).

Filters applied: Humans, English, Adult: 19+, from 2005-2023.
Results: 423

b. Embase Search Strategy
Search Query: (('stroke'.mp. OR 'cerebrovascular accident'.mp. OR ‘cerebrovascular accident'/exp OR 'transient ischemic attack'/exp OR ‘transient ischemic attack’.mp. OR ‘transient ischaemic attack’.mp. OR 'brain ischemia'/exp OR ‘brain ischemia’.mp. or ‘brain ischaemia’.mp.) AND (‘endovascular surgery’/exp OR ‘endovascular surgery’.mp. OR 'endovascular procedure'.mp. OR ‘reperfusion therapy’.mp. OR ‘reperfusion’/exp OR ‘reperfusion’.mp. OR 'thrombectomy'/exp OR ‘thrombectomy’.mp. OR ‘thrombectomy device’/exp OR ‘thrombectomy device’.mp. OR 'recanalization'/exp OR OR ‘recanalization’.mp. OR ‘recanalisation’.mp.) AND ('futile recanalization'.mp. OR ‘futile recanalisation’.mp. OR 'failed recanalization'.mp. OR ‘failed recanalisation’.mp. OR 'complete recanalization'.mp. OR ‘complete recanalisation’.mp. OR 'partial recanalization'.mp. OR ‘partial recanalisation’.mp.)).

Filters applied: Human, English Language, Adult: 18+ years, from 2005-2023.
Results: 800

c. Cochrane Library Search Strategy
Search Query: Title/Abstract/Keyword: (("Acute ischemic stroke" OR "Stroke, Ischemic" OR "Cerebrovascular Accident" OR "Transient Ischemic Attack" OR "Brain Ischemia") AND ("Thrombectomy" OR "Endovascular Procedures" OR "Reperfusion Therapy" OR "Recanalization") AND ("Futile recanalization" OR "Failed recanalization" OR "Complete recanalization" OR "Partial recanalization")).

Filters applied: English, from 2005-2023.
Results: 208

d. Other Sources
Additional sources were identified through hand-searching and Google Scholar.
Results: 12

2. Supplemental Tables

a. Supplemental Table 1: PRISMA-2020 Checklist

Section and Topic	Item #	Checklist item	Location where item is reported
**TITLE**			
Title	1	Identify the report as a systematic review.	1
**ABSTRACT**			
Abstract	2	See the PRISMA 2020 for Abstracts checklist.	1
**INTRODUCTION**			
Rationale	3	Describe the rationale for the review in the context of existing knowledge.	2
Objectives	4	Provide an explicit statement of the objective(s) or question(s) the review addresses.	2
**METHODS**			
Eligibility criteria	5	Specify the inclusion and exclusion criteria for the review and how studies were grouped for the syntheses.	4
Information sources	6	Specify all databases, registers, websites, organisations, reference lists and other sources searched or consulted to identify studies. Specify the date when each source was last searched or consulted.	2-3
Search strategy	7	Present the full search strategies for all databases, registers and websites, including any filters and limits used.	Supplemental Information
Selection process	8	Specify the methods used to decide whether a study met the inclusion criteria of the review, including how many reviewers screened each record and each report retrieved, whether they worked independently, and if applicable, details of automation tools used in the process.	4, 10
Data collection process	9	Specify the methods used to collect data from reports, including how many reviewers collected data from each report, whether they worked independently, any processes for obtaining or confirming data from study investigators, and if applicable, details of automation tools used in the process.	4, 10
Data items	10a	List and define all outcomes for which data were sought. Specify whether all results that were compatible with each outcome domain in each study were sought (e.g. for all measures, time points, analyses), and if not, the methods used to decide which results to collect.	10

Section and Topic	Item #	Checklist item	Location where item is reported
	10b	List and define all other variables for which data were sought (e.g. participant and intervention characteristics, funding sources). Describe any assumptions made about any missing or unclear information.	10
Study risk of bias assessment	11	Specify the methods used to assess risk of bias in the included studies, including details of the tool(s) used, how many reviewers assessed each study and whether they worked independently, and if applicable, details of automation tools used in the process.	10
Effect measures	12	Specify for each outcome the effect measure(s) (e.g. risk ratio, mean difference) used in the synthesis or presentation of results.	10, Table 4-6, Supplemental Information
Synthesis methods	13a	Describe the processes used to decide which studies were eligible for each synthesis (e.g. tabulating the study intervention characteristics and comparing against the planned groups for each synthesis (item #5)).	10, Table 1-3
	13b	Describe any methods required to prepare the data for presentation or synthesis, such as handling of missing summary statistics, or data conversions.	10-11
	13c	Describe any methods used to tabulate or visually display results of individual studies and syntheses.	10-11
	13d	Describe any methods used to synthesize results and provide a rationale for the choice(s). If meta-analysis was performed, describe the model(s), method(s) to identify the presence and extent of statistical heterogeneity, and software package(s) used.	10-11
	13e	Describe any methods used to explore possible causes of heterogeneity among study results (e.g. subgroup analysis, meta-regression).	10-11
	13f	Describe any sensitivity analyses conducted to assess robustness of the synthesized results.	NA
Reporting bias assessment	14	Describe any methods used to assess risk of bias due to missing results in a synthesis (arising from reporting biases).	Supplemental Information
Certainty assessment	15	Describe any methods used to assess certainty (or confidence) in the body of evidence for an outcome.	Table 4-6, Supplemental Information
**RESULTS**			

Section and Topic	Item #	Checklist item	Location where item is reported
Study selection	16a	Describe the results of the search and selection process, from the number of records identified in the search to the number of studies included in the review, ideally using a flow diagram.	3, Figure 1
	16b	Cite studies that might appear to meet the inclusion criteria, but which were excluded, and explain why they were excluded.	3, Figure 1
Study characteristics	17	Cite each included study and present its characteristics.	11, Table 1-3
Risk of bias in studies	18	Present assessments of risk of bias for each included study.	Supplemental Information
Results of individual studies	19	For all outcomes, present, for each study: (a) summary statistics for each group (where appropriate) and (b) an effect estimate and its precision (e.g. confidence/credible interval), ideally using structured tables or plots.	11, 21-29, Table 4-6, Supplemental Information
Results of syntheses	20a	For each synthesis, briefly summarise the characteristics and risk of bias among contributing studies.	21-29, Table 4-6, Supplemental Information
	20b	Present results of all statistical syntheses conducted. If meta-analysis was done, present for each the summary estimate and its precision (e.g. confidence/credible interval) and measures of statistical heterogeneity. If comparing groups, describe the direction of the effect.	11-29, Table 4-6, Supplemental Information
	20c	Present results of all investigations of possible causes of heterogeneity among study results.	NA
	20d	Present results of all sensitivity analyses conducted to assess the robustness of the synthesized results.	NA
Reporting biases	21	Present assessments of risk of bias due to missing results (arising from reporting biases) for each synthesis assessed.	NA
Certainty of evidence	22	Present assessments of certainty (or confidence) in the body of evidence for each outcome assessed.	21-29
**DISCUSSION**			
Discussion	23a	Provide a general interpretation of the results in the context of other evidence.	29-32

Section and Topic	Item #	Checklist item	Location where item is reported
	23b	Discuss any limitations of the evidence included in the review.	31-32
	23c	Discuss any limitations of the review processes used.	31-32
	23d	Discuss implications of the results for practice, policy, and future research.	29-32
**OTHER INFORMATION**			
NA	24a	Provide registration information for the review, including register name and registration number, or state that the review was not registered.	NA
	24b	Indicate where the review protocol can be accessed, or state that a protocol was not prepared.	NA
	24c	Describe and explain any amendments to information provided at registration or in the protocol.	NA
Support	25	Describe sources of financial or non-financial support for the review, and the role of the funders or sponsors in the review.	32-33
Competing interests	26	Declare any competing interests of review authors.	33
Availability of data, code and other materials	27	Report which of the following are publicly available and where they can be found: template data collection forms; data extracted from included studies; data used for all analyses; analytic code; any other materials used in the review.	33, Supplemental Information

From: [1] Page MJ, McKenzie JE, Bossuyt PM, Boutron I, Hoffmann TC, Mulrow CD, et al. The PRISMA 2020 statement: an updated guideline for reporting systematic reviews. BMJ 2021;372:n71. doi: 10.1136/bmj.n7

b. Supplemental Table 2: MOOSE Checklist

Item No	Recommendation	Reported on Page No
Reporting of background should include		
1	Problem definition	2
2	Hypothesis statement	NA
3	Description of study outcome(s)	2
4	Type of exposure or intervention used	NA
5	Type of study designs used	2
6	Study population	2
Reporting of search strategy should include		
7	Qualifications of searchers (eg, librarians and investigators)	1
8	Search strategy, including time period included in the synthesis and key words	2-3, Supplemental Information
9	Effort to include all available studies, including contact with authors	NA
10	Databases and registries searched	2-3, Supplemental Information
11	Search software used, name and version, including special features used (eg, explosion)	2-3, Supplemental Information
12	Use of hand searching (eg, reference lists of obtained articles)	2-3, Supplemental Information
13	List of citations located and those excluded, including justification	33-35, Figure 1
14	Method of addressing articles published in languages other than English	NA
15	Method of handling abstracts and unpublished studies	Figure 1
16	Description of any contact with authors	NA
Reporting of methods should include		
17	Description of relevance or appropriateness of studies assembled for assessing the hypothesis to be tested	4, 10
18	Rationale for the selection and coding of data (eg, sound clinical principles or convenience)	4, 10
19	Documentation of how data were classified and coded (eg, multiple raters, blinding and interrater reliability)	10
20	Assessment of confounding (eg, comparability of cases and controls in studies where appropriate)	NA
21	Assessment of study quality, including blinding of quality assessors, stratification or regression on possible predictors of study results	10
22	Assessment of heterogeneity	Figure 4-6, Supplemental Information
23	Description of statistical methods (eg, complete description of fixed or random effects models, justification of whether the chosen models account for predictors of study results, dose-response models, or cumulative meta-analysis) in sufficient detail to be replicated	NA

Item No	Recommendation	Reported on Page No
24	Provision of appropriate tables and graphics	Tables 1-6, Figures 1-4
Reporting of results should include		
25	Graphic summarizing individual study estimates and overall estimate	Figure 2-4, Supplemental Information
26	Table giving descriptive information for each study included	Table 1-3
27	Results of sensitivity testing (eg, subgroup analysis)	Supplemental Information
28	Indication of statistical uncertainty of findings	11
**Item No**	**Recommendation**	**Reported on Page No**
Reporting of discussion should include		
29	Quantitative assessment of bias (eg, publication bias)	Supplemental Information
30	Justification for exclusion (eg, exclusion of non-English language citations)	4, Figure 1
31	Assessment of quality of included studies	29-32, Supplemental Information
Reporting of conclusions should include		
32	Consideration of alternative explanations for observed results	32
33	Generalization of the conclusions (ie, appropriate for the data presented and within the domain of the literature review)	32
34	Guidelines for future research	29-32
35	Disclosure of funding source	33

From: Stroup DF, Berlin JA, Morton SC, et al, for the Meta-analysis Of Observational Studies in Epidemiology (MOOSE) Group. Meta-analysis of Observational Studies in Epidemiology. A Proposal for Reporting. JAMA. 2000;283(15):2008-2012. doi: 10.1001/jama.283.15.2008.

c. Supplemental Table 3: Modified Jadad Analysis for Methodological Quality

StudyID	Authors	Criteria 1	Criteria 2	Criteria 3	Criteria 4	Criteria 5	Criteria 6	Criteria 7	Criteria 8	Total
1	Baek et al.	0	0	0.5	1	0	1	1	1	4.5
2	Boisseou et al.	0	0	0	0	0	1	1	1	3
3	Bouslama et al.	0	0	0	0	0	1	1	1	3
4	Dhillon et al.	0	0	0	0	0	1	1	1	3
5	Dong et al.	0	0	0.5	1	0	1	1	1	4.5
6	Espinosa de Rueda et al.	0	0	0.5	1	0	1	1	1	4.5
7	Feng et al.	0	0	0	0	0	1	1	1	3
8	Gilberti et al.	0	0	0	0	0	1	1	1	3
9	Hassan et al.	0	0	0	0	0	1	1	1	3
10	Heitkamp et al.	0	0	0	0	0	1	1	1	3
11	Hussein et al.	0	0	0.5	1	0	1	1	1	4.5
12	Lattanzi et al.	0	0	0	0	0	1	1	1	3
13	Lee et al.	0	0	0	0	0	1	1	1	3
14	Liao et al.	0	0	0.5	1	0	1	1	1	4.5
15	Lin et al.	0	0	0	0	0	1	1	1	3
16	Linfante et al.	0	0	0	0	0	1	1	1	3
17	Mechtouff et al.	0	0	0.5	1	1	1	1	1	5.5
18	Meinel et al.	0	0	0	0	0	1	1	1	3
19	Merlino et al.	0	0	0	0	0	1	1	1	3
20	Mohammaden et al.	0	0	0	0	0	0	1	1	2
21	Ni et al.	0	0	0	0	0	1	1	1	3
22	Odezimir et al.	0	0	0	0	0	1	1	1	3

23	Ouyang et al.	0	0	0	0	0	1	1	1	3
24	Pan et al.	0	0	0.5	1	0	1	1	1	4.5
25	Pedraza et al.	0	0	0.5	1	0	1	1	1	4.5
26	Pfaff et al.	0	0	0.5	1	0	0	1	1	3.5
27	Shi et al.	0	0	1	1	0	1	1	1	5
28	Singer et al.	0	0	0	0	0	1	1	1	3
29	Su et al.	0	0	0.5	1	1	1	1	1	5.5
30	Tajima et al.	0	0	0	0	0	0	1	1	2
31	Tateishi et al.	0	0	0.5	1	0	1	1	1	4.5
32	Tonetti et al.	0	0	0.5	1	0	1	1	1	4.5
33	Vatan et al.	0	0	0.5	1	0	1	1	1	4.5
34	Wang et al. (1)	0	0	0	0	0	1	1	1	3
35	Wang et al. (2)	0	0	0	0	0	1	1	1	3
36	Xie et al.	0	0	0.5	1	0	1	1	1	4.5
37	Xu et al.	0	0	0.5	1	0	1	1	1	4.5
38	Zang et al.	0	0	0.5	1	0	1	1	1	4.5
39	Zhou et al.	0	0	0.5	1	0	1	1	1	4.5

Criteria 1: Was the study randomised? (0 = not described or no, 1 = yes)
Criteria 2: Was the method of randomisation appropriate (0 = not described or no, 1 = yes)
Criteria 3: Was the study described as being blinded? (0 = not described or no, 0.5 = single blinded 1 = double-blinded)
Criteria 4: Was the method of blinding appropriate (0 = not described or no, 1 = yes)
Criteria 5: Was there a description of withdrawals and dropouts? (0 = not described or no, 1 = yes)
Criteria 6: Was there a clear description of the inclusion/exclusion criteria? (0 = not described or no, 1 = yes)
Criteria 7: Was the method used to assess adverse events described? (0 = not described or no, 1 = yes)
Criteria 8: Was the method of statistical analysis described? (0 = not described or no, 1 = yes)

d. Supplemental Table 4: Funding Bias Scores for Studies

StudyID	Authors	Publication Bias	Funding
1	Baek et al.	2	Supported by a grant from the Korea Health Technology R&D Project through the Korea Health Industry Development Institute, funded by the Ministry of Health & Welfare, Republic of Korea
2	Boisseou et al.	0	No conflicts of interest
3	Bouslama et al.	0	No conflicts of interest
4	Dhillon et al.	0	Commissioned by the Health Quality Improvement Partnership and funded by National Health Service (NHS) England and the Welsh Government
5	Dong et al.	0	No conflicts of interest
6	Espinosa de Rueda et al.	0	No conflicts of interest
7	Feng et al.	0	No conflicts of interest
8	Gilberti et al.	0	No conflicts of interest
9	Hassan et al.	1	Supported by Medtronic, Inc.
10	Heitkamp et al.	1	An author received grants and funding from the National Institute of Health, an author received compensation from Eppdata, an author received grants and personal fees from Acandis, Cerenovus, MicroVention, Medtronic, Stryker, Phenox and Route 92, an author received grants from the German Research Foundation during the conduction of the study.
11	Hussein et al.	0	No conflicts of interest
12	Lattanzi et al.	0	No conflict of interest
13	Lee et al.	2	An author received research support from Genentech and Lumosa, and travel support for academic work from Neuravi.
14	Liao et al.	1	Supported by the Luzhou Science and Technology Plan and Gulin-The Affiliated Hospital of SouthWest Medical University Strategic Cooperation Project
15	Lin et al.	1	Supported by the National Natural Science Foundation of China, the Jiangsu Pharmaceutical Association, the Hunan Natural Science Foundation, and the Hunan innovation guidance grant of clinical medical technology
16	Linfante et al.	0	No conflicts of interest

17	Mechtouff et al.	2	Supported by the RHU MARVELOUS of Université Claude Bernard Lyon-1 within the Investissements d'Avenir program operated by the French National Research Agency and the CASDEN prize from CASDEN/Fondation de l'Avenir
18	Meinel et al.	1	Supported by Medtronic (Dublin, Ireland)
19	Merlino et al.	0	No conflicts of interest
20	Mohammaden et al.	0	No conflicts of interest
21	Ni et al.	0	No conflicts of interest
22	Odezimir et al.	0	No conflicts of interest
23	Ouyang et al.	2	Supported by the Translational Medicine and Interdisciplinary Research Joint Fund of Zhongnan Hospital of Wuhan University
24	Pan et al.	0	No conflicts of interest
25	Pedraza et al.	1	Partially funded by the Spanish Ministry of Science
26	Pfaff et al.	0	No conflicts of interest
27	Shi et al.	1	Primary author supported by National Natural Science Foundation of China, Program for New Century Excellent Talents in University of China and Fundamental Research Funds for Central Universities, Sun Yat-sen University.
28	Singer et al.	N/A	No conflicts of interest declared
29	Su et al.	0	No conflicts of interest
30	Tajima et al.	0	No conflicts of interest
31	Tateishi et al.	0	No conflicts of interest
32	Tonetti et al.	N/A	No conflicts of interest declared
33	Vatan et al.	0	No conflicts of interest
34	Wang et al. (1)	1	Supported by the Natural Science Foundation for Colleges and Universities of Anhui Province and the Excellent Young Talents Foundation for Colleges and Universities of Anhui Province
35	Wang et al. (2)	0	No conflicts of interest
36	Xie et al.	0	No conflicts of interest
37	Xu et al.	2	Supported by the National Key Research and Development Program of China

38	Zang et al.	2	Supported by the National Key R&D Program of China, National Natural Science Foundation of China, Natural Science Foundation of Guangdong Province, Guangzhou Science and Technology Planning Project, Guangdong Province Aid of Xinjiang Rural Science and Technology Development (Special Commissioner) Project, and Pilot Project of Technology Promotion and Poverty Alleviation by National Health Commission
39	Zhou et al.	2	Supported by a grant from the Stroke Prevention Project of the National Health Commission of the People's Republic of China and by the Wu Jieping Medical Foundation, the National Key R&D Program of China, National Natural Science Foundation of China, Scientific and Technological Research Project of Henan Province

0 = Low potential for bias
1 = Conflicts of interest declared relating to industry funding outside of current research publication
2 = Funded by industry
3 = High potential for bias

e. Supplemental Table 5: Outputs from Egger's Test for Publication Bias for Predictive Indicators

Outcome	Std_Eff	Coefficient [95% CI]	Standard Error	t	P > | t |	Test of H0: no small-study effects
Male	Slope	-0.51 [-0.261; 0.159]	-0.5	-0.5	0.623	0.446
Male	Bias	-0.32 [-1.192; 0.537]	-0.77	-0.77	0.446	
AF	Slope	0.45 [0.1978; 0.7104]	0.12	3.64	0.001	0.332
AF	Bias	-0.45 [-1.383; 0.710]	0.45	-0.99	0.332	
Alcohol	Slope	-0.53 [-1.574; 0.516]	0.43	-1.24	0.262	0.483
Alcohol	Bias	0.69 [-1.574; 0.296]	0.93	0.75	0.483	
CVD	Slope	0.69 [-0.351; 1.737]	0.49	1.41	0.178	0.341
CVD	Bias	-1.2 [-3.656, 1.346]	1.17	-0.98	0.341	
HTN	Slope	0.29 [0.049; 0.532]	0.12	2.45	0.02	0.243
HTN	Bias	0.54 [-0.388; 1.476]	0.46	1.19	0.243	
HL	Slope	-0.09 [-0.303; 0.118]	0.1	-0.91	0.373	0.471
HL	Bias	0.25 [-0.452; 0.950]	0.34	0.73	0.471	
DM	Slope	0.39 [0.096; 0.694]	0.15	2.69	0.011	0.345
DM	Bias	0.44 [-0.496; 1.377]	0.46	0.96	0.345	
PS/TIA	Slope	0.22 [-0.090; 0.538[	0.15	1.53	0.148	0.878
PS/TIA	Bias	0.07 [-0.951; 1.10]	0.48	0.16	0.878	
Smoking	Slope	-0.44 [-0.764; -0.123]	0.16	-2.86	0.009	0.79
Smoking	Bias	0.13 [-0.877; 1.144]	0.49	0.27	0.79	
GC	Slope	-2.17 [-3.542; -0.799]	0.53	-4.07	0.01	0.115
GC	Bias	3.55 [-1.241; 8.342]	1.86	1.9	0.115	
APU	Slope	0.26 [-0.056; 0.579]	0.13	2.01	0.091	0.338
APU	Bias	-0.55 [-1.859; 0.750]	0.53	-1.04	0.338	
ACU	Slope	0.28 [-0.296; 0.865]	0.24	1.2	0.276	0.997
ACU	Bias	0.00 [-1.955; 1.961]	0.8	0	0.997	
LAA	Slope	-0.03 [-0.582; 0.518]	0.25	-0.12	0.903	0.589
LAA	Bias	-0.47 [-2.303; 1.363]	0.85	-0.55	0.589	
CE	Slope	0.12 [-0.284; 0.525]	0.2	0.64	0.535	0.435
CE	Bias	0.60 [-1.004; 2.215]	0.76	0.8	0.435	
GA	Slope	0.44 [0.053; 0.818]	0.15	2.93	0.033	0.98
GA	Bias	-0.03 [-2.545; 2.493]	0.8	-0.03	0.98	
IVT	Slope	-0.32 [-0.543; -0.094]	0.11	-2.9	0.007	0.948
IVT	Bias	0.03 [-0.892; 0.952]	0.45	0.07	0.948	
sICH	Slope	1.31 [0.266; 2.366]	0.49	2.67	0.017	0.152
sICH	Bias	0.86 [-0.355; 2.075]	0.57	1.51	0.152	
HT	Slope	1.59 [0.957; 2.221]	0.28	5.69	0	0.093
HT	Bias	-1.42 [-3.12; 0.289]	0.75	-1.88	0.093	
Mortality	Slope	51.26 [-146.848; 249.359]	71.35	0.72	0.512	0.536
Mortality	Bias	-33.03 [-168.550; 102.493]	48.81	-0.68	0.536	

Abbreviations: AF: atrial fibrillation; CVD: cardiovascular disease; HTN: hypertension; HL: hyperlipidemia; DM: diabetes mellitus; PS/TIA: prior stroke or transient ischemic attack; GC: good collaterals; APU: antiplatelet usage; ACU: anticoagulant

usage; LAA: large artery atherosclerosis; CE: cardioembolic; GA: general anaesthesia; IVT: intravenous thrombolysis; sICH: symptomatic intracranial hemorrhage; HT: hemorrhagic transformation

f. Supplemental Table 6: Summary Effects and Heterogeneity from Meta-analysis of Discrete Predictive Markers and Outcomes Associated with Futile Recanalization

Outcome	N	n	Subgroup	Effect Measure	Summary Effects	Heterogeneity		Heterogeneity Variance Estimates			
					REDL						
					OR (95% CI)	Tests of Overall Effect	Cochran's Q	H	I2 ≤	p-value	τ2 ≤
Male	23	4690	Anterior	OR	0.78 [0.669; 0.910]	p = 0.002, z = -3.167	32.65	-	32.60%	p = 0.067	0.0416
Male	4	439	Posterior	OR	1.10 [0.668; 1.799]	p = 0.716, z = 0.364	3.29	-	8.90%	p = 0.349	0.0259
Male	7	4719	Both	OR	1.00 [0.859; 1.157]	p = 0.967, z = -0.041	7.59	-	21%	p = 0.269	0.0082
Male	4941	14	Prospective	OR	0.85 [0.74; 0.97]	p = 0.016, z = -2.405	14.64	-	11.20%	p = 0.330	0.0073
Male	4907	20	Retrospective	OR	0.90 [0.75; 1.09]	p = 0.296, z = -1.044	36.72	-	48.30%	p = 0.009	0.0722
**Male**	**34**	**9848**	**Overall**	**OR**	**0.87 [0.769; 0.973]**	**p = 0.016, z = -2.416**	**51.56**	**1.25**	**36% (95% CI: [0.0%; 60.1%])**	**p = 0.021**	**0.0355**
AF	3684	19	Anterior	OR	1.45 [1.220; 1.724]	p < 0.001, z = 4.207	24.45	-	0.00%	p = 0.141	0.0354
AF	703	4	Posterior	OR	0.92 [0.633; 1.334]	p = 0.658, z = -0.443	2.91	-	0.00%	p = 0.405	0
AF	3084	5	Both	OR	1.49 [1.261; 1.749]	p < 0.001, z = 4.207	3.05	-	26.40%	p = 0.549	0
AF	11	3250	Prospective	OR	1.57 [1.358; 1.806]	p < 0.001, z = 6.159	10.25	-	2.40%	p = 0.419	0.3353
AF	17	4221	Retrospective	OR	1.27 [1.049; 1.524]	p = 0.014, z = 2.465	21.06	-	24%	p = 0.176	0.0016
**AF**	**28**	**7471**	**Overall**	**OR**	**1.39 [1.223; 1.589]**	**p < 0.001, z = 4.976**	**36.01**	**1.155**	**25% (95% CI: [0.05%; 53.9%]**	**p = 0.115**	**0.0268**
Alcohol	6	934	Anterior	OR	0.75 [0.518; 1.077]	p = 0.119, z = -1.561	1.66	-	0%	p = 0.893	0
Alcohol	2	170	Posterior	OR	1.01 [0.581; 1.101]	p = 0.981, z = 0.024	1.72	-	41.70%	p = 0.190	0.1614

Alcohol	-	-	Both	OR	-	-	-	-	-	-	-
Alcohol	2	255	Prospective	OR	0.83 [0.44; 1.57]	p = 0.573, z = -0.563	0.11	-	0.00%	p = 0.737	0
Alcohol	6	849	Retrospective	OR	0.79 [0.55; 1.14]	p = 0.208, z = -1.260	3.8	-	0.00%	p = 0.579	0
**Alcohol**	**8**	**1104**	**Overall**	**OR**	**0.80 [0.581; 1.101]**	**p = 0.170, z = -1.372**	**3.93**	**0.75**	**0.0% (95% CI: [0.0%; 25.4%])**	**p = 0.787**	**0**
CVD	10	1637	Anterior	OR	1.17 [0.668; 2.043]	p = 0.585, z = 0.546	36.98	-	75.70%	p < 0.001	0.0902
CVD	3	225	Posterior	OR	0.83 [0.204; 1.671]	p = 0.794, z = -0.261	4.46	-	55.20%	p = 0.108	0.8383
CVD	4	748	Both	OR	1.28 [0.817; 2.011]	p = 0.281, z = 1.079	5.26	-	43.00%	p = 0.154	0.5668
CVD	1269	7	Prospective	OR	1.27 [0.656; 2.469]	p = 0.475, z = 0.714	31.08	-	80.70%	p < 0.001	0.6042
CVD	1341	10	Retrospective	OR	1.01 [0.699; 1.447]	p = 0.974, z = 0.033	12.54	-	28.30%	p = 0.184	0.0913
**CVD**	**17**	**2610**	**Overall**	**OR**	**1.15 [0.795; 1.671]**	**p = 0.454, z = 0.748**	**48.28**	**1.737**	**66.9% (95% CI: [14.6%; 82.5%])**	**p < 0.001**	**0.3634**
HTN	22	4327	Anterior	OR	1.77 [1.465; 2.148]	p < 0.001, z = 5.865	35.42	-	40.70%	p = 0.025	0.0744
HTN	4	439	Posterior	OR	1.32 [1.412; 1.924]	p = 0.216, z = 1.238	1.33	-	0.00%	p = 0.721	0
HTN	7	4672	Both	OR	1.53 [1.136; 2.059]	p = 2.802; z = 1.238	21.48	-	72.10%	p = 0.002	0.0926
HTN	4894	14	Prospective	OR	1.76 [1.37; 2.25]	p < 0.001, z = 4.480	38.12	-	65.90%	p < 0.001	0.1207
HTN	4544	19	Retrospective	OR	1.57 [1.28; 1.93]	p < 0.001, z = 4.269	29.41	-	38.80%	p = 0.044	0.0657
**HTN**	**33**	**9438**	**Overall**	**OR**	**1.65 [1.412; 1.924]**	**p < 0.001, z = 6.330**	**67.53**	**1.453**	**52.6% (95% CI: [0.5%; 72.4%])**	**p < 0.001**	**0.082**
HL	19	3674	Anterior	OR	0.96 [0.821; 1.124]	p = 0.617, z = -0.500	9.28	-	0.00%	p = 0.953	0
HL	3	225	Posterior	OR	0.99 [0.354; 2.749]	p = 0.980, z = -0.-25	4.53	-	55.90%	p = 0.104	0.4508

HL	6	2537	Both	OR	1.02 [0.809; 1.272]	p = 0.900, z = 0.125	6.81	-	26.60%	p = 0.235	0.0212
HL	10	2086	Prospective	OR	1.10 [0.851; 1.409]	p = 0.480, z = 0.707	11.55	-	22.10%	p = 0.240	0.0356
HL	18	4350	Retrospective	OR	0.935 [0.820; 1.064]	p = 0.308, z = -1.020	7.92	-	0.00%	p = 0.968	0
**HL**	**28**	**6436**	**Overall**	**OR**	**0.97 [0.870; 1.088]**	**p = 0.627, z = -0.486**	**20.84**	**0.878**	**0.0% (95% CI: [0.0%; 21.1%])**	**p = 0.794**	**0**
DM	22	4326	Anterior	OR	1.78 [1.519; 2.080]	p < 0.001, z = 7.176	13.8	-	0.00%	p = 0.878	0
DM	4	439	Posterior	OR	1.54 [0.805; 2.963]	p = 0.191, z = 1.306	5.51	-	45.60%	p = 0.138	0.2116
DM	7	4670	Both	OR	1.61 [1.066; 2.425]	p = 0.024, z = 2.265	28.34	-	78.80%	p < 0.001	0.1984
DM	4892	14	Prospective	OR	1.99 [1.613; 2.446]	p < 0.001, z = 6.464	17.85	-	27.20%	p = 0.163	0.0382
DM	4543	19	Retrospective	OR	1.49 [1.198; 1.842]	p < 0.001, z = 3.606	27.9	-	35.50%	p = 0.064	0.069
**DM**	**33**	**9435**	**Overall**	**OR**	**1.71 [1.468; 1.990]**	**p < 0.001, z = 6.912**	**48.81**	**1.235**	**34.4% (95% CI: [0.0%; 58.9%])**	**p = 0.029**	**0.057**
PS/TIA	9	1639	Anterior	OR	1.53 [1.139; 2.051]	p = 0.005, z = 2.825	2.89	-	0.00%	p = 941	0
PS/TIA	3	225	Posterior	OR	1.33 [0.262; 6.750]	p = 0.730, z = 0.345	7.12	-	71.90%	p = 0.028	1.46
PS/TIA	4	3956	Both	OR	1.09 [0.793; 1.508]	p = 0.585, z = 0.546	6.03	-	50.30%	p = 0.110	0.0471
PS/TIA	2567	5	Prospective	OR	1.34 [1.07; 1.70]	p = 0.01, z = 2.59	4.24	-	5.80%	p = 0.374	0.0053
PS/TIA	3253	11	Retrospective	OR	1.14 [0.80; 1.62]	p = 0.48, z = 0.71	14.33	-	30.20%	p = 0.159	0.0943
**PS/TIA**	**16**	**4820**	**Overall**	**OR**	**1.30 [1.058; 1.592]**	**p = 0.012, z = 2.502**	**18.7**	**1.117**	**19.8% (95% CI: [0.0%; 57.3%])**	**p = 0.227**	**0.0287**
Smoking	17	3101	Anterior	OR	0.65 [0.535; 0.783]	p < 0.001, z = -4.82	18.73	-	14.60%	p = 0.283	0.0224
Smoking	4	439	Posterior	OR	0.81 [0.370; 1.779]	p = 0.603, z = -0.521	9.32	-	67.80%	p = 0.025	0.422
Smoking	4	2055	Both	OR	0.70 [0.566; 0.854]	p = 0.001, z = -3.468	0.55	-	0.00%	p = 0.907	0.0005

Smoking	8	1659	Prospective	OR	0.68 [0.49; 0.95]	p = 0.024, z = -5.351	13.12	-	46.60%	p = 0.069	0.0992
Smoking	17	3936	Retrospective	OR	0.66 [0.57; 0.77]	p < 0.001, z = -2.259	15.88	-	0.00%	p = 0.561	0
**Smoking**	**25**	**5595**	**Overall**	**OR**	**0.66 [0.572; 0.772]**	**p < 0.001, z = -5.349**	**29.01**	**1.099**	**17.3% (95% CI: [0.0%; 50.0%])**	**p = 0.220**	**0.0227**
GC	7	1925	Anterior	OR	0.33 [0.225; 0.486]	p < 0.001, z = -5.632	17.59	1.712	65.9% (95% CI: [0.0%; 86.4%])	p = 0.007	0.1659
GC	-	-	Posterior	OR	-	-	-	-	-	-	-
GC	-	-	Both	OR	-	-	-	-	-	-	-
GC	3	874	Prospective	OR	0.29 [0.198; 0.425]	p < 0.001, z = -6.351	2.82	-	29.10%	p = 0.244	0.0336
GC	4	1051	Retrospective	OR	0.42 [0.21; 0.86]	p = 0.018, z = -2.370	14.75	-	79.70%	p = 0.002	0.3905
**GC**	**7**	**1925**	**Overall**	**OR**	**0.33 [0.225; 0.486]**	**p < 0.001, z = -5.632**	**17.59**	**1.712**	**65.9% (95% CI: [0.0%; 86.4%])**	**p = 0.007**	**0.1659**
APU	4	1027	Anterior	OR	1.13 [0.846; 1.511]	p = 0.407, z = 0.829	3	-	0.00%	p = 0.392	3.89e^-7
APU	1	84	Posterior	OR	1.16 [0.976; 1.368]	p = 0.694, z = -0.394	0	-	0.00%	-	0
APU	3	1824	Both	OR	1.18 [0.955; 1.453]	p = 0.125, z = 1.533	0.62	-	-	p = 0.735	0
APU	1908	4	Retrospective	OR	1.17 [0.949; 1.438]	p = 0.142, z = 1.467	0.97	-	0.00%	p = 0.809	0
APU	1027	4	Prospective	OR	1.13 [0.846; 1.511]	p = 0.407; z = 0.829	3	-	0.00%	p = 0.392	3.89e-07
**APU**	**8**	**2935**	**Overall**	**OR**	**1.16 [0.976; 1.386]**	**p = 0.094, z = 1.676**	**4**	**0.756**	**0.0% (95% CI: [0.0%; 33.0%])**	**p = 0.779**	**0**
ACU	4	919	Anterior	OR	1.47 [0.914; 2.351]	p = 0.113, z = 1.586	5.27	-	43.10%	p = 0.153	0.0984
ACU	1	84	Posterior	OR	0.65 [0.103; 4.104]	p = 0.647, z = -0.458	0	-	-	-	0
ACU	3	1824	Both	OR	1.3 [0.998; 1.679]	p = 0.051, z = 1.948	0.61	-	0.00%	p = 0.738	0
ACU	4	2203	Retrospective	OR	1.36 [1.074; 1.729]	p = 0.011, z = 2.551	2.79	-	0.00%	p = 0.594	0
ACU	3	624	Prospective	OR	1.31 [0.717; 2.381]	p - 0.382, z = 0.874	3.72	-	46.20%	p = 0.156	0.13005

**ACU**	**8**	**2827**	**Overall**	**OR**	**1.33 [1.083; 1.634]**	**p = 0.007, z = 2.716**	**6.67**	**0.97 6**	**0.0% (95% CI: [0.0%; 54.1%])**	**p = 0.464**	**0**
LAA	9	1875	Anterior	OR	0.75 [0.553; 1.008]	p = 0.056, = -1.911	16.17	-	50.50%	p = 0.040	0.099 1
LAA	3	384	Posterior	OR	1.00 [0.620; 1.595]	p = 0.983, z = -0.021	0.73	-	0.00%	p = 0.693	0
LAA	3	1824	Both	OR	0.97 [0.661; 1.418]	p = 0.867, z = -0.167	2.5	-	19.90%	p = 0.287	0.031
LAA	6	1222	Prospectiv e	OR	0.80 [0.61; 1.04]	p = 0.093, z = -1.682	5.67	-	11.80%	p = 0.340	0.013 7
LAA	9	2861	Retrospect ive	OR	0.82 [0.60; 1.12]	p = 0.217, z = -1.234	15.73	-	49.10%	p = 0.046	0.098 6
**LAA**	**15**	**4083**	**Overall**	**OR**	**0.83 [0.671; 1.018]**	**p = 0.073, z = -1.793**	**22.02**	**1.26 4**	**36.5% (95% CI: [0.0; 66.7%])**	**p = 0.078**	**0.054 9**
CE	11	2633	Anterior	OR	1.50 [1.166; 1.932]	p = 0.002, z = 3.156	21.76	-	54%	p = 0.016	0.091 5
CE	3	384	Posterior	OR	0.99 [0.441; 2.219]	p = 0.980, z = -0.025	4.13	-	51.60%	p = 0.127	0.265 2
CE	3	1824	Both	OR	1.16 [0.958; 1.398]	p = 0.131, z = 1.511	0.45	-	0.00%	p = 0.798	0
CE	7	1685	Prospectiv e	OR	1.41 [1.004; 1.988]	p = 0.047, z = 1.984	14.42	-	58.40%	p = 0.025	0.117 5
CE	10	3156	Retrospect ive	OR	1.27 [1.012; 1.581]	p = 0.039, z = 2.069	14.31	-	37.10%	p = 0.112	0.041
**CE**	**17**	**4841**	**Overall**	**OR**	**1.34 [1.100; 1.625]**	**p = 0.003, z = 3.016**	**31.36**	**1.4**	**49% (95% CI: [0.0%; 73.3%])**	**p = 0.012**	**0.067 1**
GA	4	1331	Anterior	OR	1.55 [1.352; 1.737]	p = 0.002, z = 3.121	4.02	-	25.40%	p = 0.259	0.02
GA	-	-	Posterior	OR	-	-	-	-	-	-	-
GA	3	3922	Both	OR	1.53 [1.305; 1.791]	p < 0.001, z = 5.267	2.64	-	24.30%	p = 0.267	0.005
GA	5	3469	Prospectiv e	OR	1.57 [1.312; 1.882]	p < 0.001, z = 4.905	5.08	-	21.20%	p = 0.280	0.009
GA	2	1784	Retrospect ive	OR	1.42 [1.352; 1.737]	p < 0.001, z = 3.568	0.6	-	0.00%	p = 0.440	0
**GA**	**7**	**5253**	**Overall**	**OR**	**1.53 [1.352; 1.737]**	**p < 0.001, z = 6.673**	**6.67**	**1.05 5**	**10.1% (95% CI: [0.0%; 62.8%])**	**p = 0.352**	**0.003 1**

IVT	20	4441	Anterior	OR	0.78 [0.644; 0.937]	p = 0.008, z = -2.642	35.04	-	45.80%	p = 0.014	0.075
IVT	4	439	Posterior	OR	0.87 [0.570; 1.332]	p = 0.524, z = -0.637	1.99	-	0.00%	p = 0.574	0
IVT	6	4485	Both	OR	0.66 [0.586; 0.750]	p < 0.001, z = -6.527	4.02	-	0.00%	p = 0.547	0
IVT	12	4755	Prospective	OR	0.76 [0.603; 0.962]	p = 0.022, z = -2.286	27.18	-	59.50%	p = 0.004	0.0869
IVT	18	4610	Retrospective	OR	0.746 [0.648; 0.860]	p < 0.001, z = -4.060	18.7	-	9.10%	p = 0.346	0.0082
IVT	30	9365	Overall	OR	0.75 [0.662; 0.857]	p < 0.001, z = -4.310	46.1	1.261	37.1% (95% CI: [0.0%; 62.0%])	p = 0.023	0.0381
sICH	10	2153	Anterior	OR	7.93 [3.952; 15.892]	p < 0.001, z = 5.832	12.57	-	28.40%	p = 0.183	0.3289
sICH	2	170	Posterior	OR	12.89 [4.889; 11.116]	p = 0.016, z = 2.417	0.02	-	0.00%	p = 0.877	0
sICH	5	2311	Both	OR	7.41 [3.790; 14.482]	p < 0.001, z = 5.856	2.29	-	0.00%	p = 0.683	0
sICH	7	2797	Prospective	OR	8.81 [4.84; 16.05]	p < 0.001, z = 7.113	4.76	-	0.00%	p = 0.575	0
sICH	10	1837	Retrospective	OR	6.65 [3.60; 12.31]	p < 0.001, z = 6.037	9.78	-	8.00%	p = 0.369	0.0815
**sICH**	**17**	**4634**	**Overall**	**OR**	**7.37 [4.889; 11.116]**	**p < 0.001, z = 9.533**	**15.18**	**0.974**	**0.0% (95% CI: [0.0%; 41.6%])**	**p = 0.511**	**0**
HT	9	1946	Anterior	OR	3.06 [2.431; 3.857]	p < 0.001, z = 9.503	8.29	-	3.50%	p = 0.406	0.0046
HT	1	55	Posterior	OR	0.81 [0.141; 4.648]	p = 0.814, z = -0.235	0	-	-	-	0
HT	1	97	Both	OR	2.92 [0.888; 9.575]	p = 0.078, z = 1.765	0	-	-	p = 0.398	0
HT	4	713	Prospective	OR	2.97 [1.958; 4.517]	p < 0.001, z = 5.112	3.77	-	20.40%	p = 0.288	0.0413
HT	7	1385	Retrospective	OR	2.86 [2.107; 3.879]	p < 0.001, z = 6.744	6.43	-	6.70%	p = 0.377	0.0118
**HT**	**11**	**2098**	**Overall**	**OR**	**2.98 [2.374; 3.746]**	**p < 0.001, z = 9.389**	**10.5**	**1.024**	**4.7% (95% CI: [0.0%; 53.5%])**	**p = 0.398**	**0.0073**
Mortality	4	837	Anterior	OR	53.74 [12.856; 224.653]	p < 0.001, z = 5.459	1.36	-	0.00%	p = 0.716	0

Mortality	-	-	Posterior	OR	-	-	-	-	-	-	-
Mortality	1	97	Both	OR	160.52 [9.120; 2825.241]	p = 0.001, z = 3.471	0	-	-	-	0
Mortality	130	1	Prospective	OR	31.65 [1.82; 549.70]	p = 0.018, z = 2.372	0	-	-	-	0
Mortality	804	4	Retrospective	OR	80.66 [19.263; 337.738]	p < 0.001, z = 6.009	1.47	-	0.0%	p = 0.688	0
**Mortality**	**5**	**934**	**Overall**	**OR**	**66.83 [118.581; 240.36]**	**P < 0.001, z = 6.434**	**1.80**	**0.671**	**0.0% (95% CI: [0.0%; 20.4%])**	**p = 0.772**	**0**

Abbreviations: AF: atrial fibrillation; CVD: cardiovascular disease; HTN: hypertension; HL: hyperlipidemia; DM: diabetes mellitus; PS/TIA: prior stroke or transient ischemic attack; GC: good collaterals; APU: antiplatelet usage; ACU: anticoagulant usage; LAA: large artery atherosclerosis; CE: cardioembolic; GA: general anaesthesia; IVT: intravenous thrombolysis; sICH: symptomatic intracranial hemorrhage; HT: hemorrhagic transformation; N: number of studies; n: number of patients; OR: odds ratio; CI: confidence interval; REDL: DerSimonian and Larid random effects method; Q: heterogeneity measures were calculated from data with 95% confidence intervals (95% CI), based on noncentral X^2 (common effect) distribution for Cochran's Q test; H: relative excess in Cochran's Q over its degrees of freedom; I^2: proportion of total variation in effect estimate due to between study heterogeneity (based on Cochran's Q test); τ^2: between-study variance to test comparisons of heterogeneity among subgroups; *: values of I≤ are percentages; ¶:heterogeneity values were calculated from data with 95% CIs based on gamma (random effects) distribution for Q; Φ: heterogeneity variance estimates (τ^2≤) were derived from the DerSimonian and Laird method

g. Supplemental Table 7: Summary Effects and Heterogeneity from Meta-analysis of Continuous Predictive Markers Associated with Futile Recanalization

Outcome	Total Number of Studies	Total Number of Patients	Occlusion Location	Effect Measure	Summary Effects REDL	Heterogeneity		Heterogeneity Variance Estimates			
					SMD (95% CI)	Tests of Overall Effect	Cochran's Q	H	I2 ≤	p-value	τ2
Age	22	4625	Anterior	SMD	0.55 [0.465; 0.627]	p < 0.0001, z = 13.244	33.91	-	38.10%	p = 0.037	0.0128
Age	4	439	Posterior	SMD	0.14 [-0.112, 0.395]	p < 0.274, z = 1.093	4.31	-	30.40%	p = 0.230	0.0204
Age	5	2343	Both	SMD	0.46 [0.324; 0.593]	p < 0.0001, z = 6.673	6.97	-	42.60%	p = 0.137	0.0096
Age	12	2724	Prospective	SMD	0.45 [0.336; 0.571]	p < 0.001, z = 7.576	22.77	-	51.70%	p = 0.019	0.0208
Age	19	4693	Retrospective	SMD	0.52 [0.42; 0.61]	p < 0.001, z = 10.400	35.7	-	49.60%	p = 0.008	0.0191
**Age**	**31**	**7417**	**Overall**	**SMD**	**0.49 [0.417; 0.564]**	**p < 0.0001, z = 13.033**	**59.67**	**1.41**	**49.7% (95% CI: [2.7%; 69.3%])**	**p = 0.001**	**0.0186**
SBP	12	2491	Anterior	SMD	0.25 [0.166, 0.327]	p < 0.001, z = 6.025	5.65	-	0% (95% CI: [0.00%, 0.00%])	p = 0.895	0
SBP	2	298	Posterior	SMD	-0.15 [-0.398; 0.095]	p = 0.229, z = -1.204	0.01	-	0% (95% CI: [0.00%, 0.00%])	p = 0.935	0
SBP	4	2052	Both	SMD	0.20 [0.046; 0.352]	p = 0.011, z = 2.551	5.28	-	43.20%	p = 0.153	0.0104
SBP	8	1794	Prospective	SMD	0.23 [0.096; 0.358]	p = 0.001, z = 3.407	12.03	-	41.80%	p = 0.100	0.014
SBP	10	3047	Retrospective	SMD	0.16 [0.083; 0.229]	p < 0.001, z = 4.203	7.24	-	0.00%	p = 0.613	0
**NIHSS**	**18**	**4841**	**Overall**	**SMD**	**0.20 [0.127; 0.266]**	**p < 0.001, z = 5.538**	**20.86**	**1.11**	**18.5% (95% CI: 0.0%; 54.5%)**	**p = 0.233**	**0.0039**
NIHSS	20	4479	Anterior	SMD	0.80 [0.657; 0.935]	p < 0.001, z = 11.206	79.41	-	76.10%	p < 0.001	0.1729
NIHSS	6	4188	Posterior	SMD	0.74 [0.107; 1.373]	p < 0.001, z = 8.798	9.23	-	78.30%	p = 0.01	0.0694

NIHSS	3	225	Both	SMD	0.62 [0.480; 0.754]	p = 0.022, z = 2.293	16.66	-	70%	p = 0.005	0.2431
NIHSS	12	4636	Prospective	SMD	0.63 [0.51; 0.76]	p < 0.001, z = 11.491	34.97	-	68.50%	p < 0.001	0.0294
NIHSS	17	4256	Retrospective	SMD	0.84 [0.70; 0.98]	p < 0.001, z = 9.764	59.67	-	73.20%	p < 0.001	0.056
**NIHSS**	**29**	**8892**	**Overall**	**SMD**	**0.75 [0.648; 0.857]**	**p < 0.001, z = 14.088**	**124.87**	**2.122**	**77.6% (95% CI: [42.9%, 88.1%])**	**p < 0.001**	**0.0535**
ASPECTS	17	3410	Anterior	SMD	-0.39 [-0.512; -0.257]	p < 0.001, z = -5.919	46.57	-	65.60%	p < 0.001	0.4178
ASPECTS	1	55	Posterior	SMD	-0.64 [-1.290; 0.012]	p = 0.054, z = -1.923	0	-	-	-	0
ASPECTS	4	2123	Both	SMD	-0.29 [-0.382; -0.206]	p < 0.001, z = -6.566	2.52	-	0% (95% CI: [0.00%, 0.00%])	p = 0.471	0
ASPECTS	10	2280	Prospective	SMD	-0.38 [-0.54; -0.22]	p < 0.001, z = -4.734	27.86	-	67.70%	p = 0.001	0.0401
ASPECTS	12	3308	Retrospective	SMD	-0.36 [-0.49; -0.23]	p < 0.001, z = -5.431	24.13	-	54.40%	p = 0.012	0.0228
**ASPECTS**	**22**	**5588**	**Overall**	**SMD**	**-0.37 [-0.464; -0.271]**	**p < 0.001, z = -7.471**	**52.01**	**1.574**	**59.6% (95% CI: [3.4%; 78.0%])**	**p < 0.001**	**0.0265**
OTT	10	2231	Anterior	SMD	0.19 [0.088; 0.285]	p < 0.001, z = 3.723	11.11	-	19%	p = 0.268	0.0046
OTT	3	353	Posterior	SMD	0.13 [-0.345; 0.613]	p = 0.583, z = 0.549	7	-	71.40%	p = 0.030	0.1248
OTT	5	4380	Both	SMD	0.29 [0.129; 0.444]	p < 0.001, z = 3.571	19.33	-	79.30%	p = 0.001	0.0229
OTT	9	3835	Prospective	SMD	0.29 [0.16; 0.42]	p < 0.001, z = 4.242	21.9	-	63.50%	p = 0.005	0.0231
OTT	9	3129	Retrospective	SMD	0.14 [0.03; 0.25]	p = 0.009, z = 2.628	11.98	-	33.20%	p = 0.152	0.0074
**OTT**	**18**	**6964**	**Overall**	**SMD**	**0.22 [0.131; 0.304]**	**p < 0.001, z = 4.925**	**38.5**	**1.505**	**55.8% (95% CI: [0.0%; 77.7%])**	**p = 0.002**	**0.0153**
OTR	11	1824	Anterior	SMD	0.33 [0.057; 0.609]	p = 0.018, z = 2.367	72.99	-	86.30%	p < 0.001	0.175
OTR	3	225	Posterior	SMD	0.42 [0.195; 0.748]	p = 0.001, z = 3.345	1.24	-	76%	p = 0.537	0

OTR	3	667	Both	SMD	0.42 [0.080; 0.764]	p = 0.016, z = 2.420	8.34	-	0% (95% CI: [0.00%, 0.00%])	p = 0.015	0.0759
OTR	7	995	Prospective	SMD	0.26 [0.05; 0.47]	p = 0.016, z = 2.407	25.49	-	76.50%	p < 0.001	0.058
OTR	10	1721	Retrospective	SMD	0.48 [0.16; 0.79]	p = 0.003, z = 2.976	46.51	-	80.60%	p < 0.001	0.1994
**OTR**	**17**	**2716**	**Overall**	**SMD**	**0.38 [0.185; 0.565]**	**p < 0.001, z = 3.863**	**83.57**	**2.285**	**80.9% (95% CI: [46.8%; 90.2%])**	**p < 0.001**	**0.1182**
BG	12	3147	Anterior	SMD	0.30 [0.180; 0.428]	p < 0.001, z = 4.817	28.34	-	61.20%	p = 0.003	0.0265
BG	2	225	Posterior	SMD	0.13 [-0.144; 0.402]	p = 0.354, z = 0.928	0.63	-	0.00%	p = 0.730	0
BG	3	1727	Both	SMD	0.45 [0.350; 0.546]	p < 0.001, z = 8.974	0.38	-	0.00%	p = 0.535	0
BG	9	2720	Prospective	SMD	0.31 [0.171; 0.46]	p < 0.001, z = 4.293	23.89	-	66.50%	p = 0.002	0.0291
BG	8	2379	Retrospective	SMD	0.32 [0.18; 0.45]	p < 0.001, z = 4.537	11.24	-	37.70%	p = 0.129	0.013
**BG**	**17**	**5099**	**Overall**	**SMD**	**0.31 [0.217; 0.409]**	**p < 0.001, z = 6.367**	**35.91**	**1.498**	**55.4% (95% CI: [0.0%; 77.1%])**	**p = 0.003**	**0.0191**

Abbreviations: SBP: systolic blood pressure; NIHSS: National Institute of Health Stroke Severity; ASPECTS: Alberta Stroke Program Early CT Score; OTT: onset-to-treatment time; OTR: onset-to-recanalization time; BG: blood glucose; N: number of studies; n: number of patients; OR: odds ratio; CI: confidence interval; REDL: DerSimonian and Larid random effects method; Q: heterogeneity measures were calculated from data with 95% confidence intervals (95% CI), based on noncentral X^2 (common effect) distribution for Cochran's Q test; H: relative excess in Cochran's Q over its degrees of freedom; I^2: proportion of total variation in effect estimate due to between study heterogeneity (based on Cochran's Q test); τ^2: between-study variance to test comparisons of heterogeneity among subgroups; *: values of I≤ are percentages; ¶:heterogeneity values were calculated from data with 95% CIs based on gamma (random effects) distribution for Q; $^{\Phi}$: heterogeneity variance estimates (τ^2≤) were derived from the DerSimonian and Laird method

3. Supplemental Figures:

a. Supplemental Figure 1: Forest Plots of Discrete Predictive Indicators of Futile Recanalization, Stratified by Occlusion Location (1)

Association between Sex and Futile Recanalization

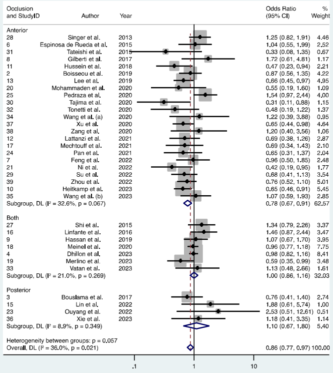

Association between AF and Futile Recanalization

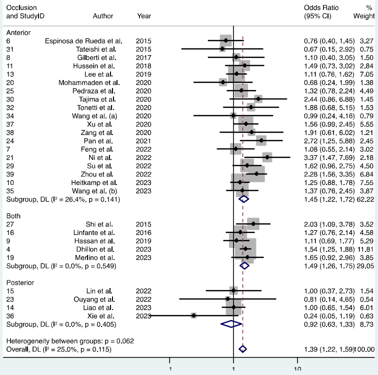

Association between Alcohol and Futile Recanalization

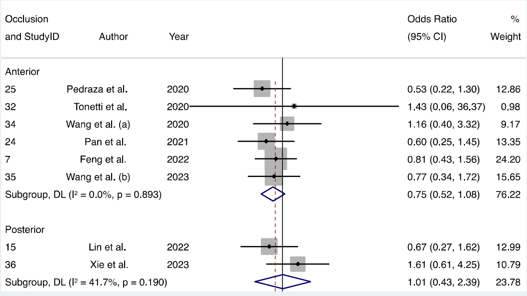

Association between CAD and Futile Recanalization

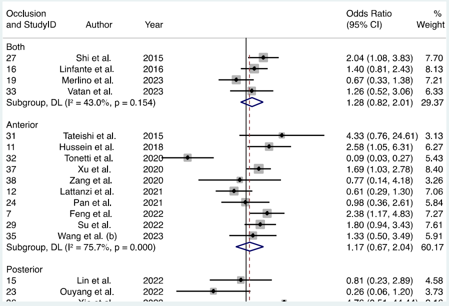

Association Between Hypertension and Futile Recanalization

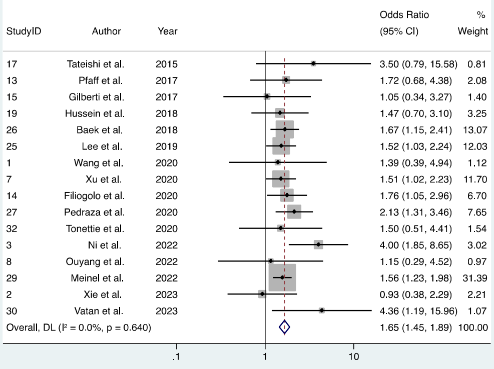

Association Between Hyperlipidemia and Futile Recanalization

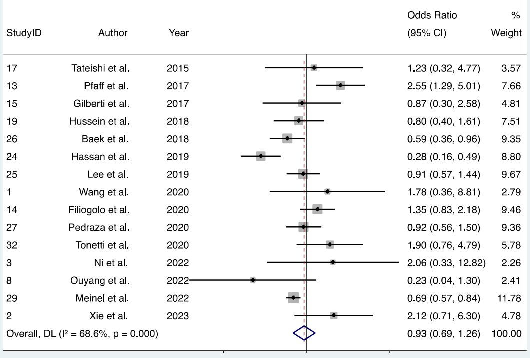

Association between DM and Futile Recanalization

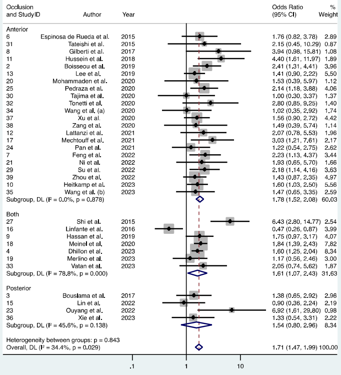

Association Between Previous Stroke/TIA and Futile Recanalization

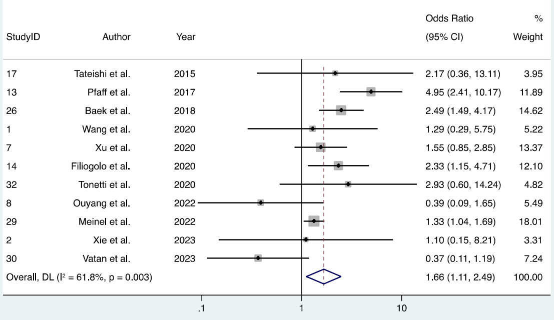

b. Supplemental Figure 2: Forest Plots of Discrete Predictive Indicators of Futile Recanalization, Stratified by Occlusion Location (2)

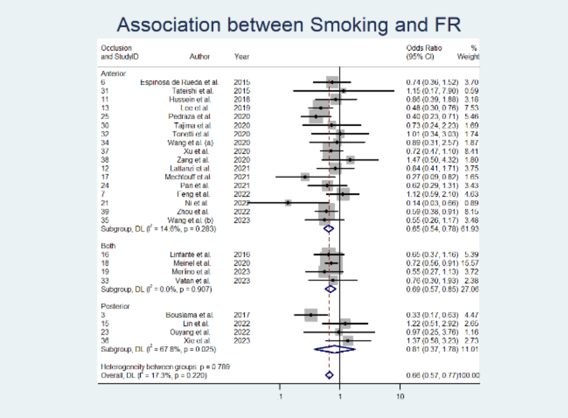

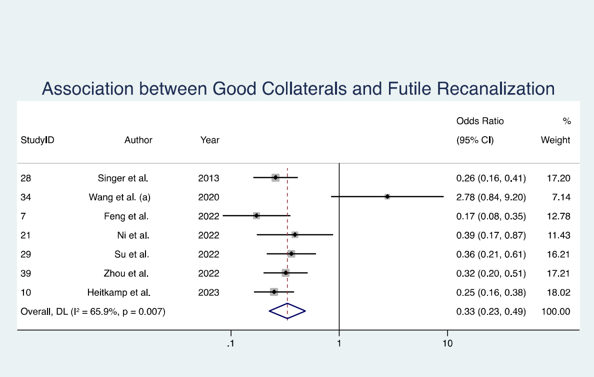

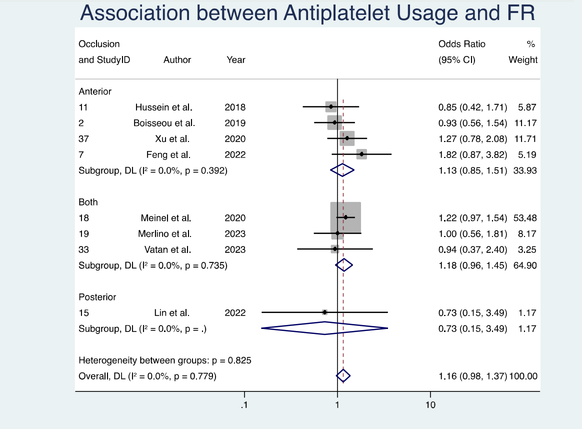

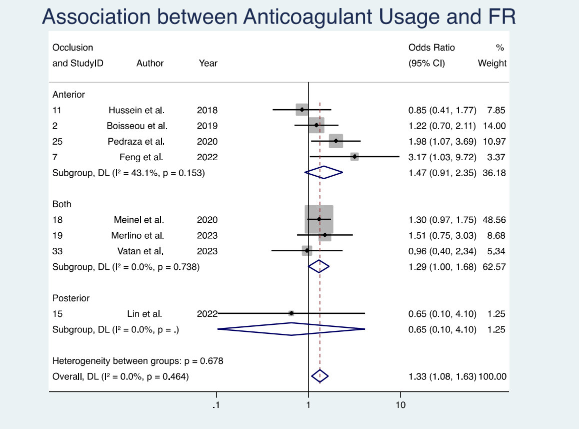

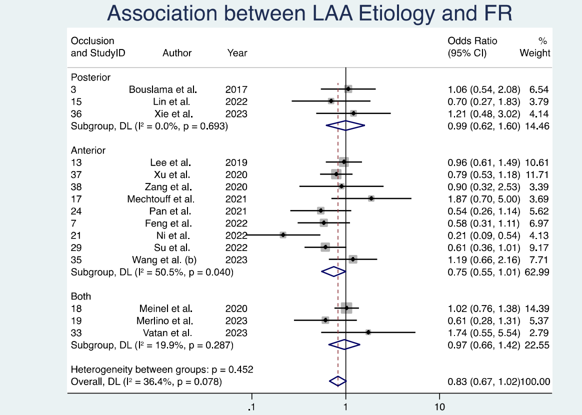

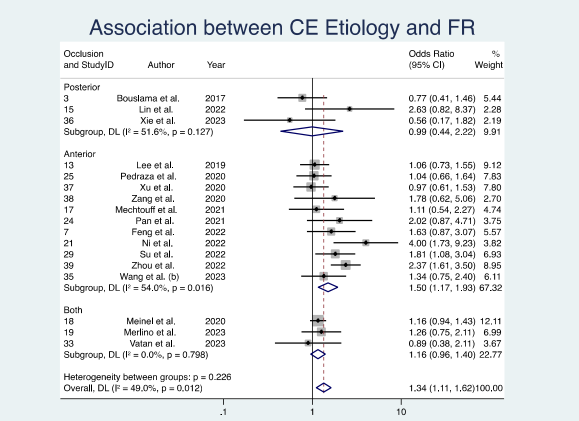

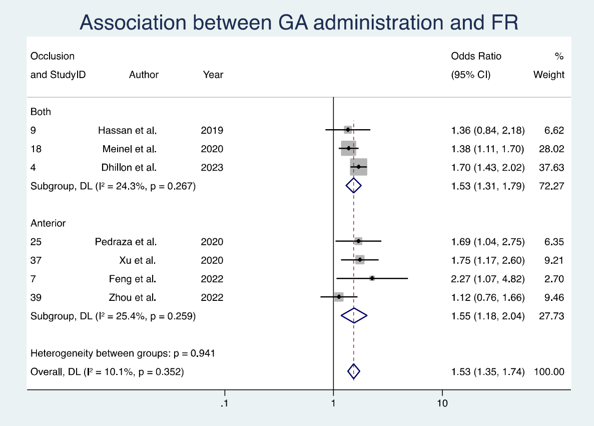

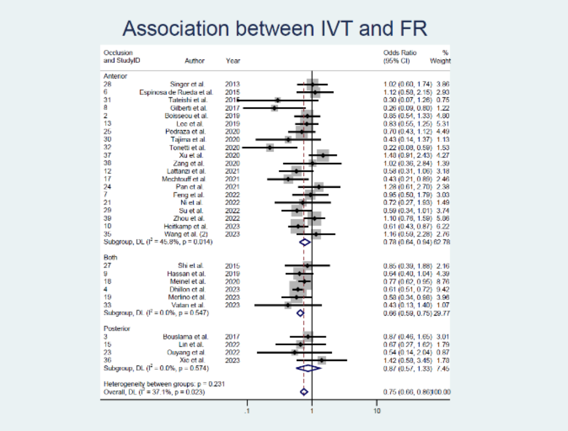

c. Supplemental Figure 3: Forest Plots of Continuous Predictive Indicators of Futile Recanalization, Stratified by Occlusion Location

Association between Age and Futile Recanalization

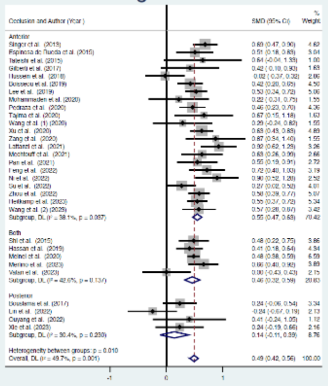

Association between Baseline SBP and Futile Recanalization

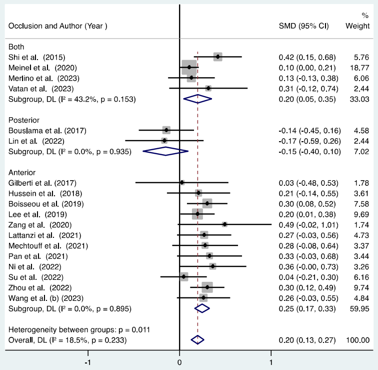

Association between Baseline NIHSS Score and Futile Recanalizat

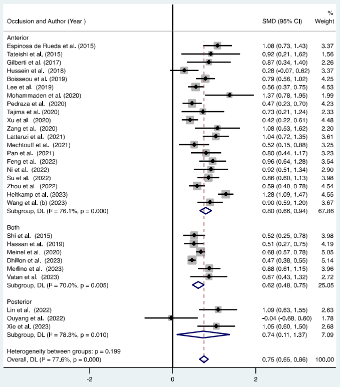

Association between Baseline ASPECTS and Futile Recanalization

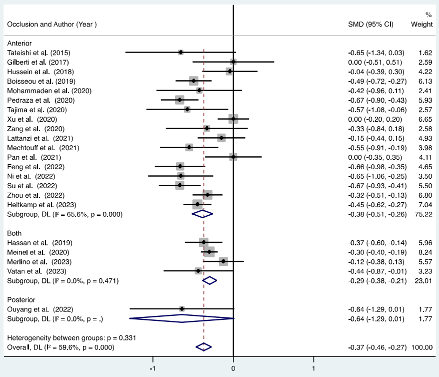

Association between OTT and Futile Recanalization

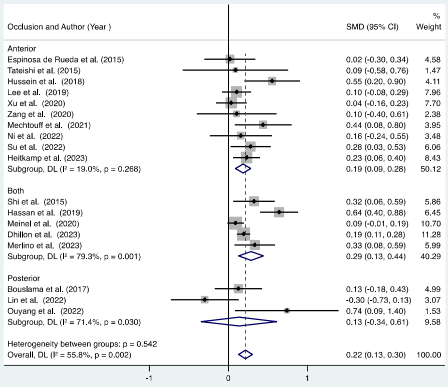

Association between OTR and Futile Recanalization

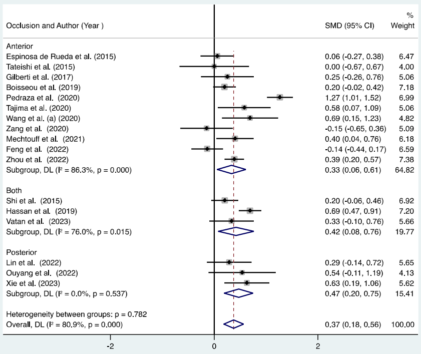

Association between baseline BG and Futile Recanalization

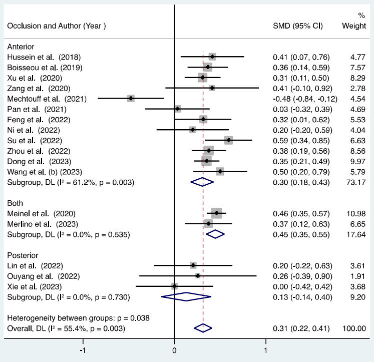

d. Supplemental Figure 4: Forest Plots of Discrete Predictive Indicators of Futile Recanalization, Stratified by Study Design (1)

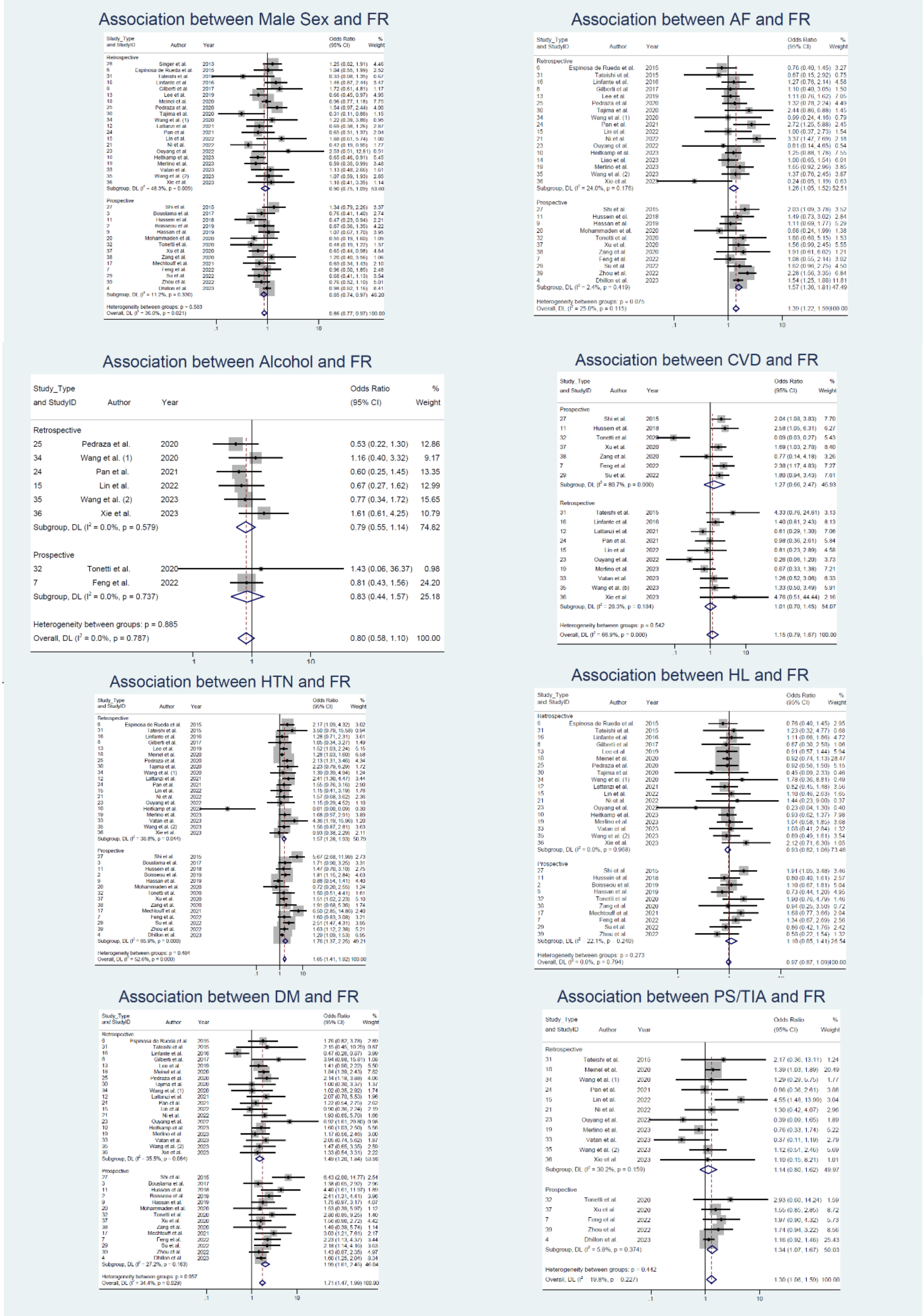

e. Supplemental Figure 5: Forest Plots of Discrete Predictive Indicators of Futile Recanalization, Stratified by Study Design (2)

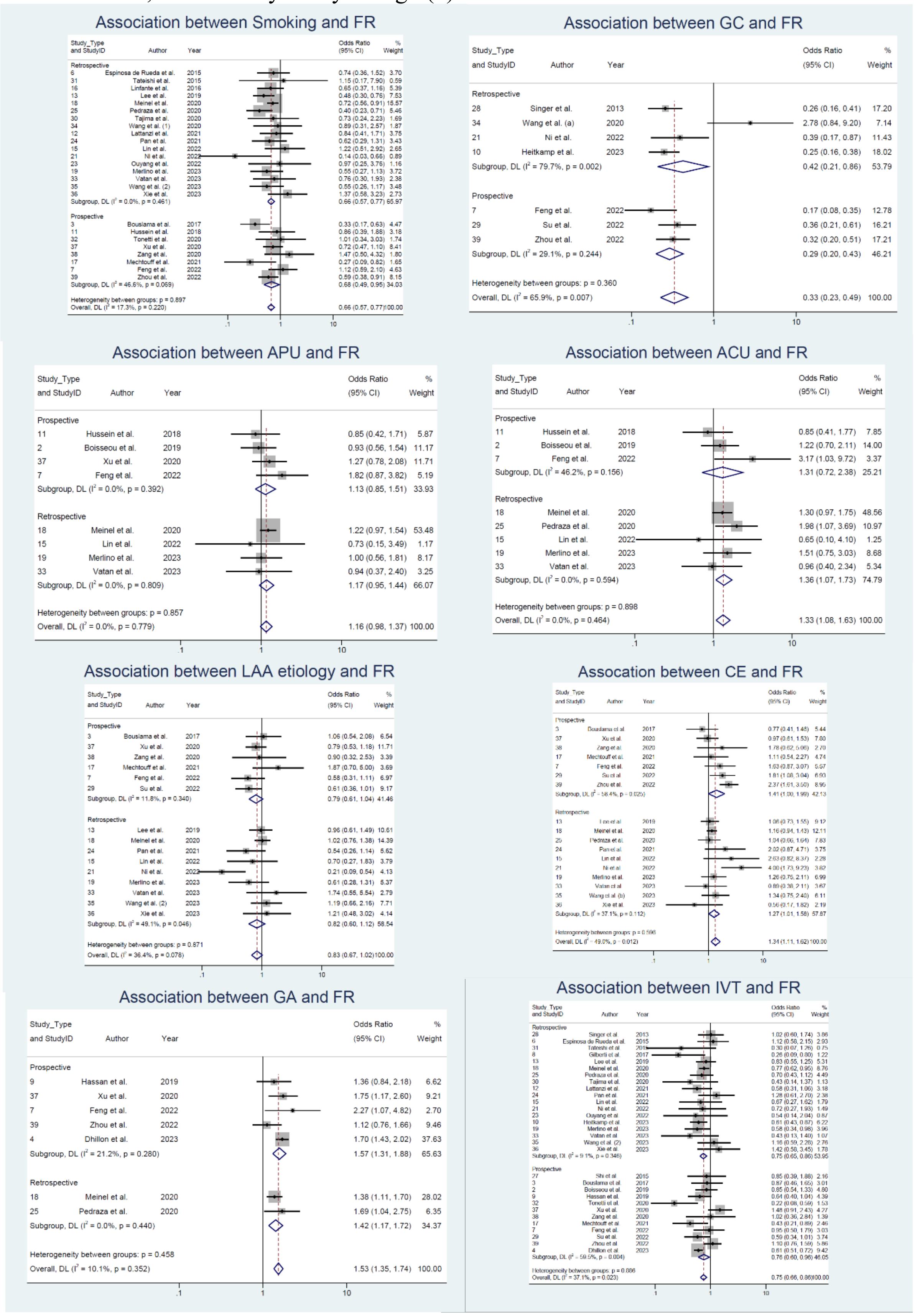

f. Supplemental Figure 6: Forest Plots of Continuous Predictive Indicators of Futile Recanalization, Stratified by Study Design

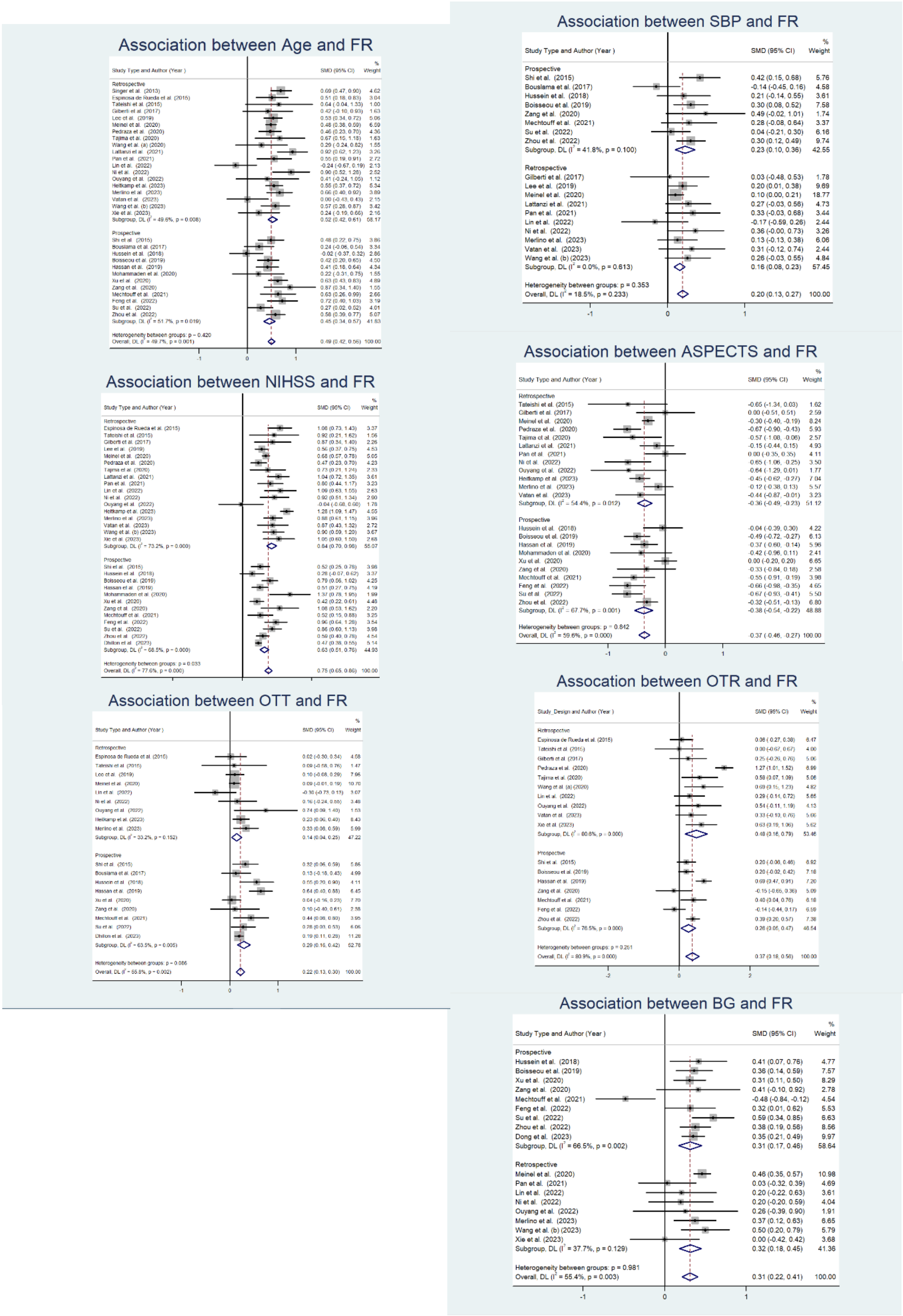

g. Supplemental Figure 7: Graphs of Egger's Regression Test for Meta-analysis on the Association between Predictive Indicators and Futile Recanalization

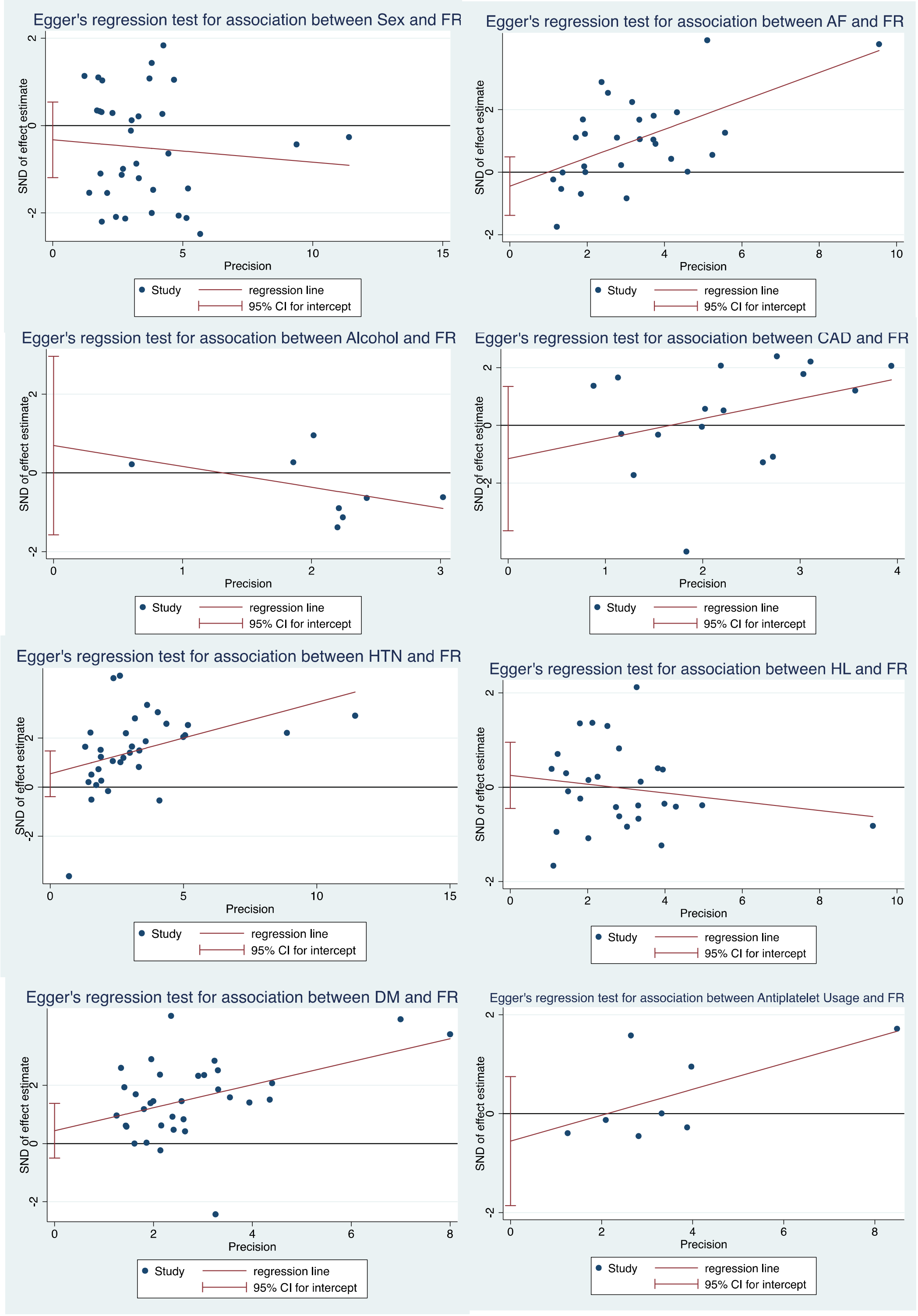

h. Supplemental Figure 8: Graphs of Egger's Regression Test for Meta-analysis on the Association between Predictive Indicators and Futile Recanalization (2)

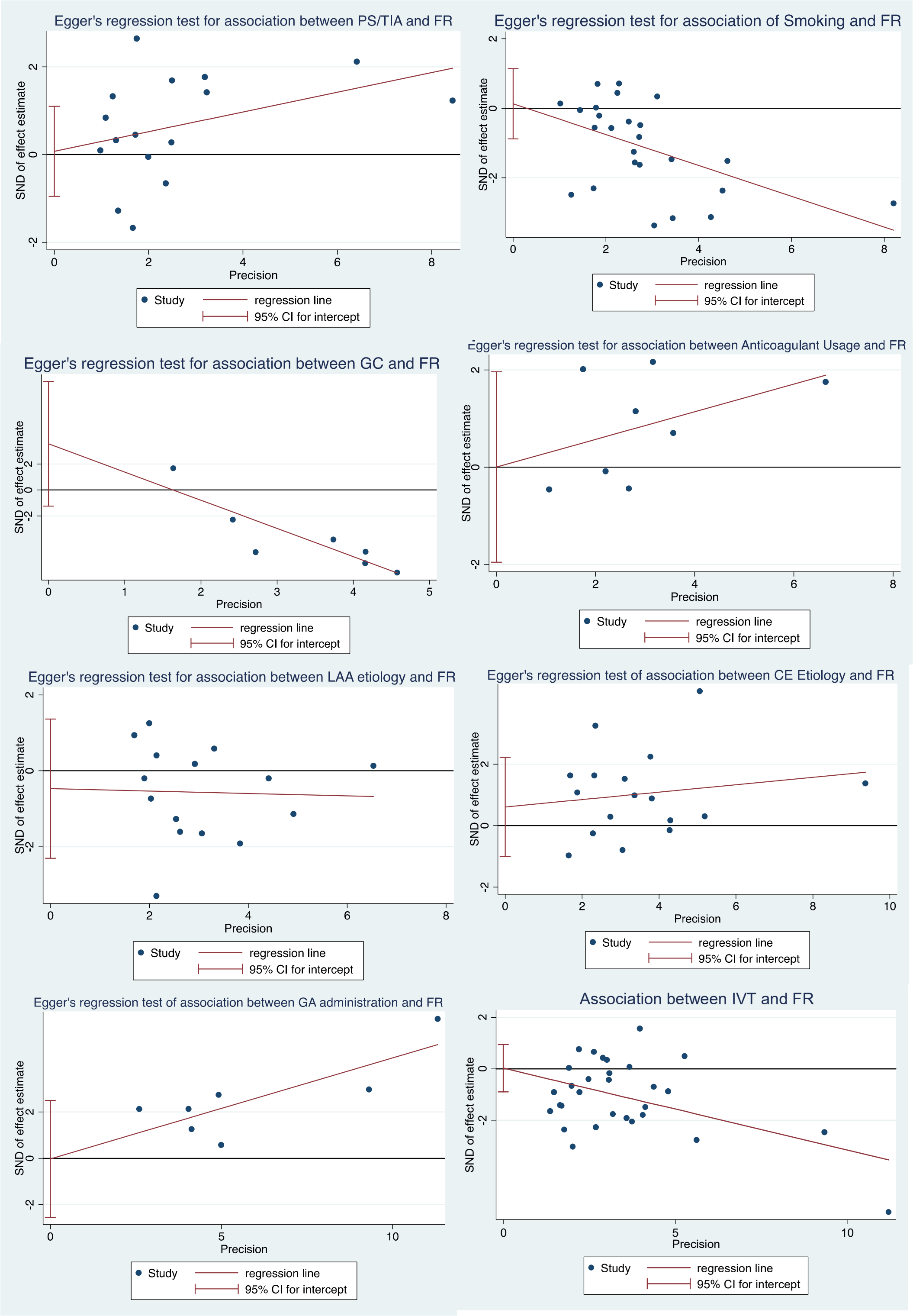

i. Supplemental Figure 9: Graphs of Egger's Regression Test for Meta-analysis on the Association between Clinical Outcomes and Futile Recanalization

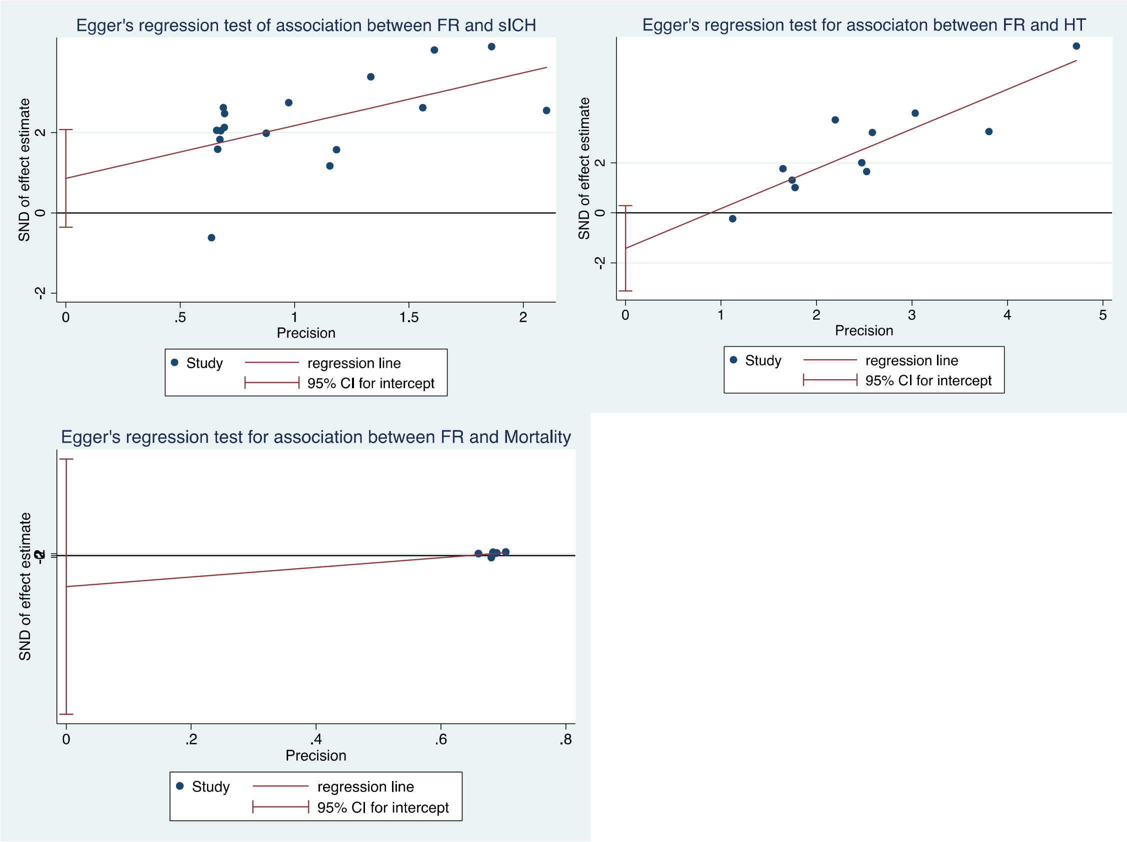

j. Supplemental Figure 10: Sensitivity Analysis on Association between Predictive Indicators and Futile Recanalization (1)

(a) Male sex

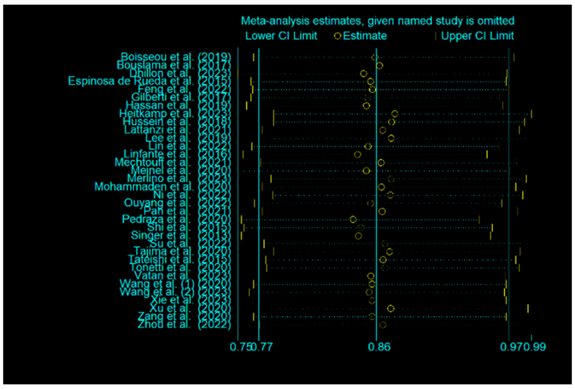

(b) Atrial fibrillation

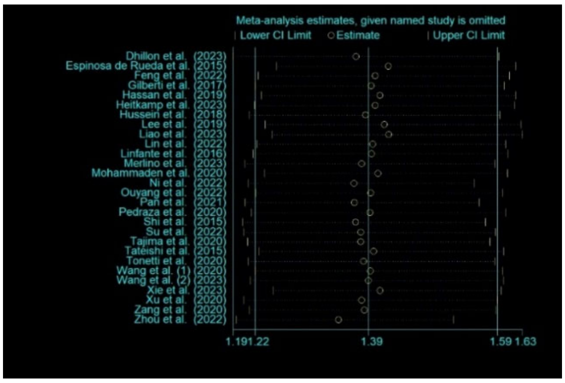

(c) Alcohol

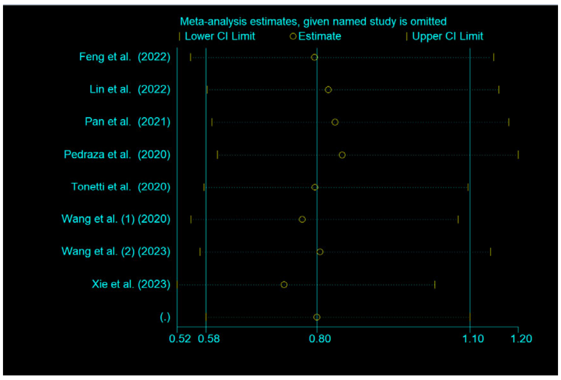

(d) Cardiovascular disease

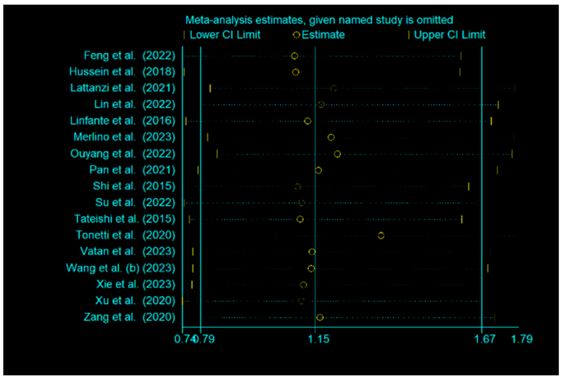

(e) Hypertension

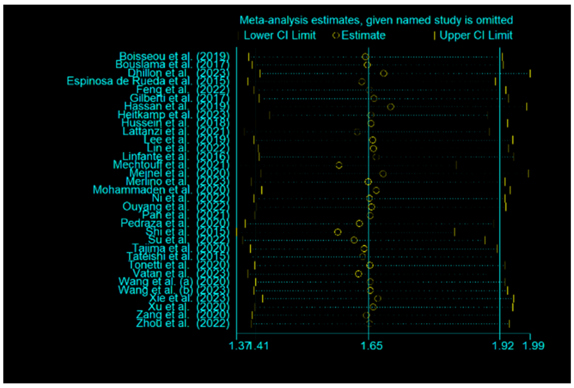

(f) Hyperlipidemia

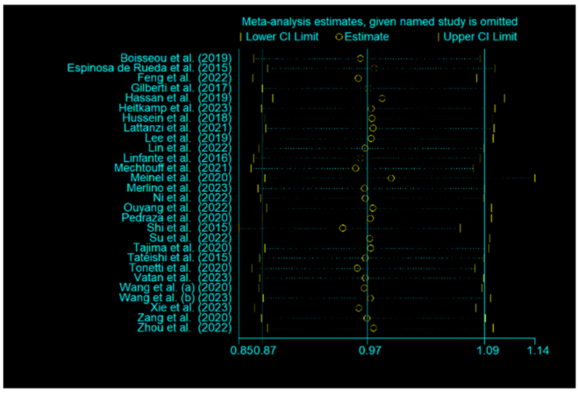

k. Supplemental Figure 11: Sensitivity Analysis on Association between Predictive Indicators and Futile Recanalization (2)

(g) Diabetes mellitus

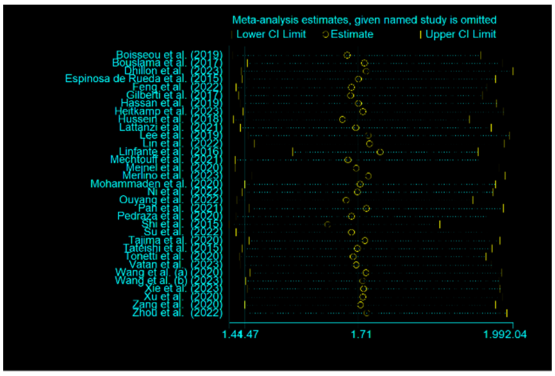

(h) Prior stroke/transient ischemic attack

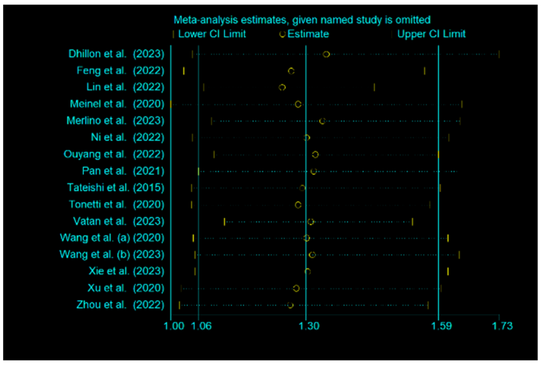

(i) Smoking

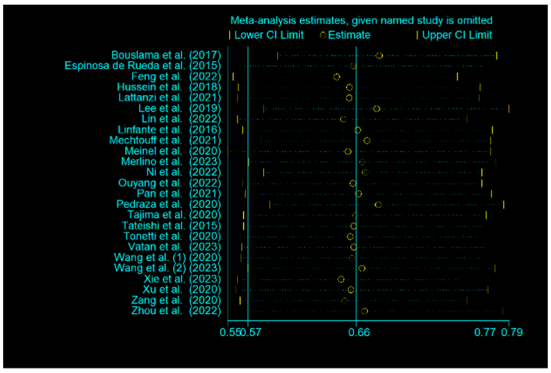

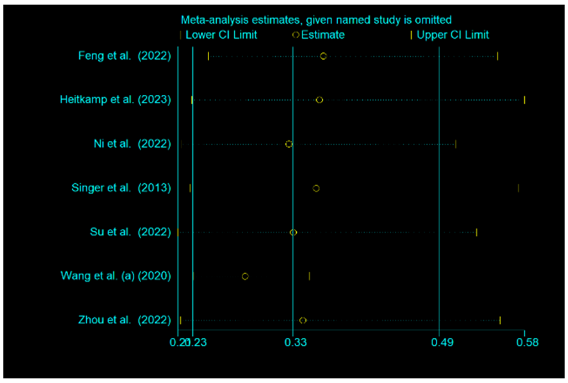

(k) Antiplatelet usage

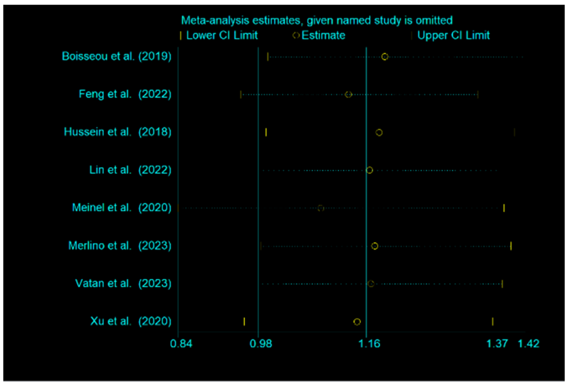

(l) Anticoagulant usage

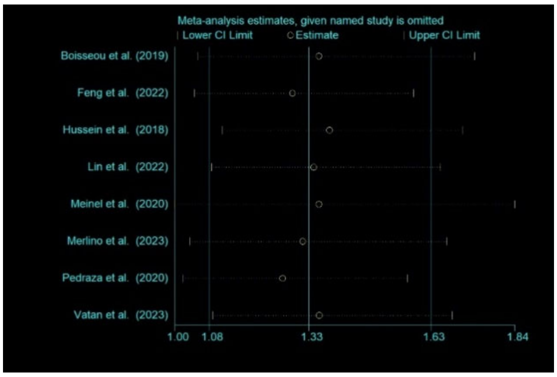

(j) Good collaterals

I. Supplemental Figure 12: Sensitivity Analysis on Association between Predictive Indicators and/or Outcomes and Futile Recanalization

(m) Large artery atherosclerosis etiology

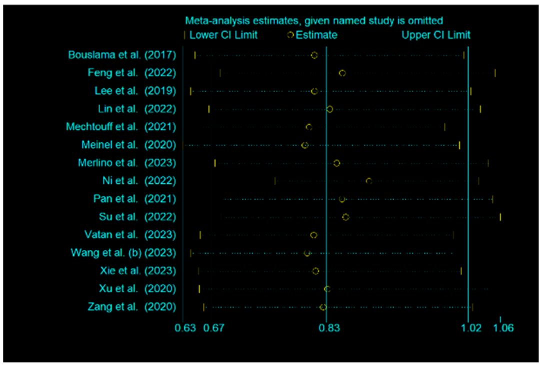

(n) Cardioembolic etiology

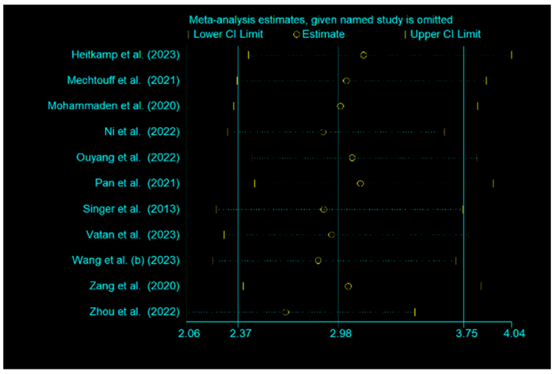

(r) Mortality

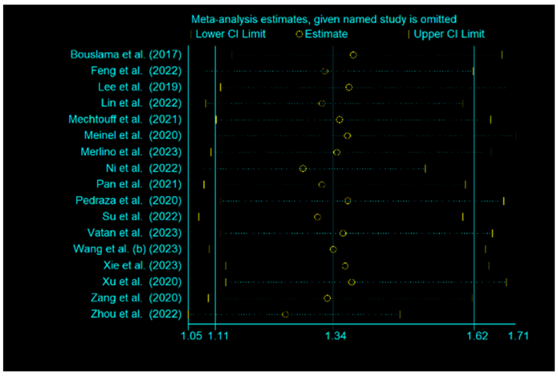

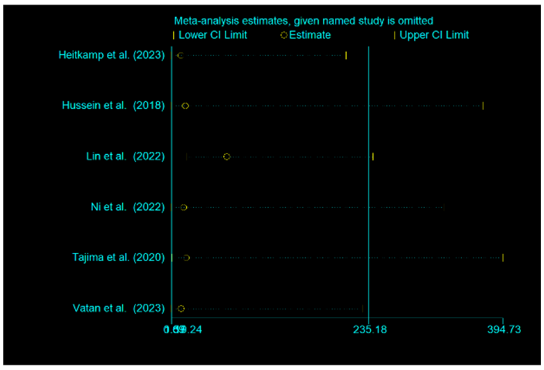

(o) Intravenous thrombolysis

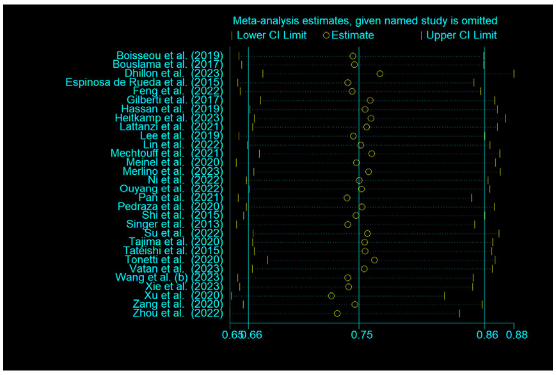

(p) Symptomatic intracranial hemorrhage

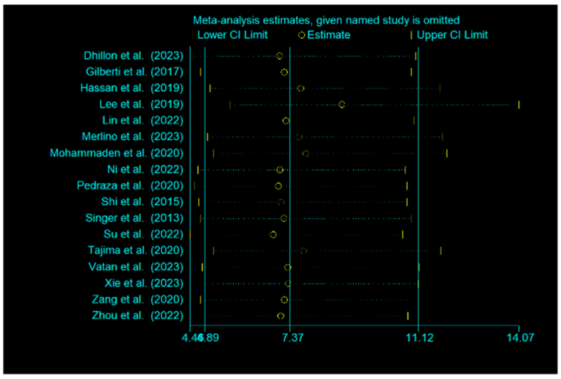

(q) Hemorrhagic transformation
